# Refgenie: a reference genome resource manager

**DOI:** 10.1093/gigascience/giz149

**Published:** 2020-01-29

**Authors:** Michał Stolarczyk, Vincent P Reuter, Jason P Smith, Neal E Magee, Nathan C Sheffield

**Affiliations:** 1 Center for Public Health Genomics, University of Virginia, PO Box 800717, Charlottesville, VA, 22908, USA; 2 Department of Biochemistry and Molecular Genetics, University of Virginia, PO Box 800733, Charlottesville, VA, 22908, USA; 3 Research Computing, University of Virginia, 560 Ray C. Hunt Drive, Charlottesville, VA, 22903, USA; 4 Department of Public Health Sciences, University of Virginia, PO Box 800717, Charlottesville, VA, 22908, USA; 5 Department of Biomedical Engineering, University of Virginia, PO Box 400259, Charlottesville, VA, 22904, USA

**Keywords:** reference genomes, reference assemblies, data management, data portability

## Abstract

**Background:**

Reference genome assemblies are essential for high-throughput sequencing analysis projects. Typically, genome assemblies are stored on disk alongside related resources; *e.g.*, many sequence aligners require the assembly to be indexed. The resulting indexes are broadly applicable for downstream analysis, so it makes sense to share them. However, there is no simple tool to do this.

**Results:**

Here, we introduce refgenie, a reference genome assembly asset manager. Refgenie makes it easier to organize, retrieve, and share genome analysis resources. In addition to genome indexes, refgenie can manage any files related to reference genomes, including sequences and annotation files. Refgenie includes a command line interface and a server application that provides a RESTful API, so it is useful for both tool development and analysis.

**Conclusions:**

Refgenie streamlines sharing genome analysis resources among groups and across computing environments. Refgenie is available at https://refgenie.databio.org.

## Background

Enormous effort goes into assembling and curating reference genomes [1–5]. These reference assemblies provide a common representation for comparing results, and they form the basis for a wide range of downstream tools for sequence alignment and annotation. Many tools that rely on reference assemblies will produce independent resources that accompany an assembly. For instance, many aligners must hash the genome, creating indexes that are used to improve alignment performance [6–9].

Analytical pipelines typically rely on these aligners and their indexes for the initial steps of a data analysis. These assembly resources are typically shared among many pipelines, so it is common for a research group to organize them in a central folder to prevent duplication. In addition to saving disk space, centralization simplifies sharing software that uses a reference assembly because software can be written around a standard folder structure. However, this does not solve the problem of sharing genomic resources between research groups. Because each group may use a different strategy to identify shared genome resources, sharing tools across groups may require modifying them.

One solution to this problem is to have a web-accessible server where standard, organized reference assemblies are available for download. Indeed, this is exactly the goal of Illumina’s iGenomes project, which provides “a collection of reference sequences and annotation files for commonly analyzed organisms” [10]. The iGenomes project has become a popular source of genome assets and has greatly simplified sharing analysis tools among research environments. However, this approach is hampered by some fundamental drawbacks and leaves several challenges unsolved. First, the individual assets can only be downloaded in bulk, but what if a particular use case requires only a small subset of resources in a package? More important, building the resources is not scripted, so if the repository excludes a reference or resource of interest, there is no programmatic way to fill the gap. In these scenarios, users must manually build and organize genome assets individually, forfeiting the strength of standardization among groups.

To improve the ability to share interoperable reference genome assets, we have developed refgenie, which enables a more modular, customizable, and user-controlled approach to managing reference assembly resources. Like iGenomes, refgenie standardizes reference genome asset organization so software can be built around that organization. But unlike iGenomes, refgenie also automates the building of genome assets, so that an identical representation can be produced for any genome assembly. Furthermore, refgenie allows programmatic access to individual resources both remote and local, making it suitable for the next generation of self-contained pipelines.

Refgenie can organize any files that can be assigned to a particular reference genome assembly, which could include not only genome indexes but other resource types such as genome sequences and annotations [11–13].

Refgenie manages genome-related resources flexibly. It can handle any asset type, from annotations to indexes. It provides individual, pre-built asset downloads from a server and allows scripted building for custom inputs. Refgenie thus solves a major hurdle in biological data analysis.

## Results and Discussion

**Figure 1:**
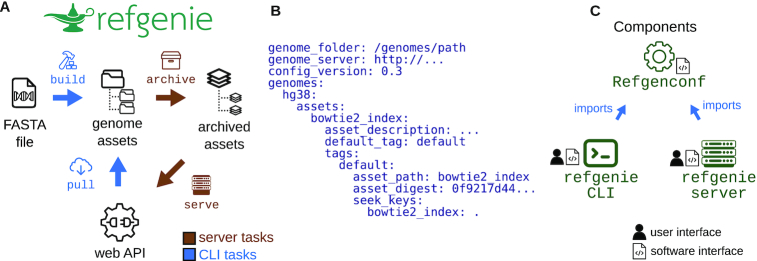
Refgenie concept and software organization. A, Refgenie provides the ability to either build or pull assets. B, Genome configuration file. Refgenie reads and writes a genome configuration file in YAML format to keep track of available local assets. C, Refgenie is tripartite, made up of a conf utility, a command line interface (CLI), and a server package. The configuration package is intended for programmatic use and is used by the CLI and server packages. Users and software use refgenie via the CLI or server (web API).

Refgenie is the first full-service reference genome asset manager. Refgenie provides 2 ways to obtain genome assets: *pull* and *build* (Fig. 1A). For common assets, pulling a pre-built, remote-hosted asset obviates the need to install and run specialized software to build a particular asset. It also makes it easier to satisfy prerequisites programmatically for pipelines and other software. However, remote-hosted assets are only practical for common genomes and assets, so for uncommon assets or on unconnected computers, users may instead *build* assets, which creates the same standard output for custom genomes. By providing both *build* and *pull*, refgenie facilitates asset organization both within and between research groups, increasing interoperability of tools that rely on genome resources.

### Asset organization

Refgenie uses a local YAML file called the “genome configuration file" (Fig. 1B) to keep track of metadata, such as local file paths. In this file, refgenie stores paths to individual genome assembly resources, or “assets," each of which represents one or more files. One can think of a genome asset as a folder of related files tied to a particular genome assembly. For example, an asset could be an index for a particular tool, or a group of annotation files. Refgenie assets are referred to using “asset registry paths," which are human-readable asset identifiers. The registry path follows the structure {genome}/{asset}:{tag}; a genome thus operates as a sort of namespace for a set of assets, which are identified both by asset names as well as by tags, allowing refgenie to manage multiple versions of the same asset.

Therefgenie software suite allows users to interact with assets with 3 components: (i) a command line interface (CLI), (ii) a server, and (iii) a configuration package that supports them both (Fig. 1C).

### Refgenie command line interface

The workhorse of refgenie is the CLI; it is how users will typically interact with genome assets. Its implementation as a command line tool not only makes it useful for general purpose exploration and access but also allows it to be integrated into existing workflows that require access to genome assets from the shell. The CLI can be installed withpip install refgenie and invoked by calling refgenie. The refgenie CLI provides 7 functions for interacting with local genome assets:

- refgenie init—initializes an empty genome configuration file
- refgenie list—summarizes the genome configuration file, listing local genomes and assets
- refgenie seek—provides the file path to a given asset
- refgenie add—adds an already-built local asset
- refgenie remove—removes a local asset
- refgenie tag—adds a tag to a local asset
- refgenie build—builds a new asset

#### Initializing refgenie

All of the CLI commands require knowledge of the refgenie configuration file, which is passed via the -cargument. To install and configure refgenie requires only a few lines of code:

pip install --user refgenie

export REFGENIE='refgenie.yaml'

refgenie init -c $REFGENIE

In this example, we populate the $REFGENIEenvironment variable, which eliminates the need to pass -c to each command going forward. The init, list, add, and remove functions are relatively straightforward and simply allow a user to create, view, and manipulate the genome configuration file.

#### Building assets

**Figure 2:**
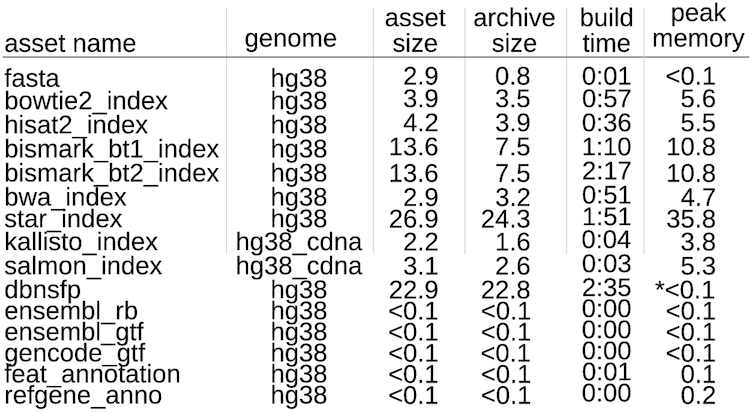
Assets available for build. Table listing assets that can currently be built with refgenie build, along with statistics for size, build time, and memory high water mark. Assets were built for the human genome using a single core. Times (in H:MM) and memory/disk (in gigabytes) are representative values from a single run. These assets are produced by various tools [8,9,14–17] and are available to be built for any arbitrary genome input. * peak disk space usage for dbnsfp is >300 GB.

The build function allows a user to *build*assets for any arbitrary inputs, which is what enables refgenie to serve custom genomes. Refgenie has built-in capability to build a selection of different common genome assets (Fig. 2). The list of assets with available recipes is displayed by the refgenie list command. Available assets are built by specifying the asset registry path along with any required inputs. For example:

refgenie build hg38/ASSET \

--files NAME=FILE

Where ASSET is a unique key defining the asset of interest (e.g., bowtie2_index),NAME is an identifier for a required input file, and FILE is a path to the provided input file. For example, to build afasta asset requires a compressed fasta file as input. It can thus be built like this:

refgenie build hg38/fasta \

--files fasta=hg38.fa.gz

 Building an asset can require either input arguments, such as in this example, or it can require other assets. The list of requirements for building an asset can be found by adding the--requirements argument to the build function. Assets are built with locally available versions of the software (e.g., bowtie2-build to create the Bowtie2 index) or alternatively with containerized software (using the -d/--docker flag in the refgenie buildcommand). We have also produced a complete containerized computing environment capable of building all available refgenie assets, which can be deployed with the bulker environment manager [18], making it easy to build any refgenie assets without installing the required tools natively.

#### Pulling assets

In addition to functions on local assets, the refgenie CLI also contains additional commands that can interact with remote assets: *pull* and *listr*:

- refgenie listr—lists available remote genomes and assets
- refgenie pull—downloads a remote asset

With these commands, refgenie downloads a standard asset with a single line of code:

refgenie pull hg38/ASSET

#### Tagging assets

The tag command allows users to tag assets with unique identifiers. Tags may also be provided when building or pulling assets to specify a version (e.g., build hg38/ASSET:TAG). Once tagged, specific versions of assets can be accessed by tag. If no tag is specified, refgenie will use the tag default, which is automatically given to any built or pulled assets that do not specify a tag. This makes tags an optional feature of refgenie that are only necessary if a user desires multiple versions of the same asset.

#### Seeking assets

Once the asset has been added to refgenie via either pulling or building, the user can retrieve the path to it with refgenie seek:

refgenie seek hg38/ASSET

This command returns the file path to the specified asset for the specified genome. The seek command is portable, eliminating the need to hard-code paths or pass them as arguments. Consequently, in a pipeline or other software that requires access to genome assembly assets, the path to the localbowtie2_index asset can be retrieved with a shell command:

bowtie2_index_path=\

$(refgenie seek hg38/bowtie2_index)

### Refgenieserver

The listr and pull functions require the CLI to interact with a server. The CLI uses a configurable URL to retrieve a remote archived tarball. After retrieving the tarball, the CLI will unpack it into the appropriate folder location and update the configuration file to provide access to its path viarefgenie seek.

To support this remote function, we have developed a containerized, portable, open-source companion application called refgenieserver. Many users of refgenie will not have to be aware of the server application; however, interested users can use refgenieserver to host their own genome asset server. For example, a tool developer may wish to simplify use by hosting indexes for common reference assemblies.

Running refgenieserver is simple for users who are already familiar with refgenie. It reads the same genome configuration file format as the CLI. In fact, refgenieserver operates on the same genome configuration file and asset folders that refgenie itself builds or downloads. The server software comes with an archive command that prepares a refgenie genome folder for serving. It compresses each asset into an individual tarball. This simple system makes it easy for users to run a server using their refgenie assets.

**Figure 3:**
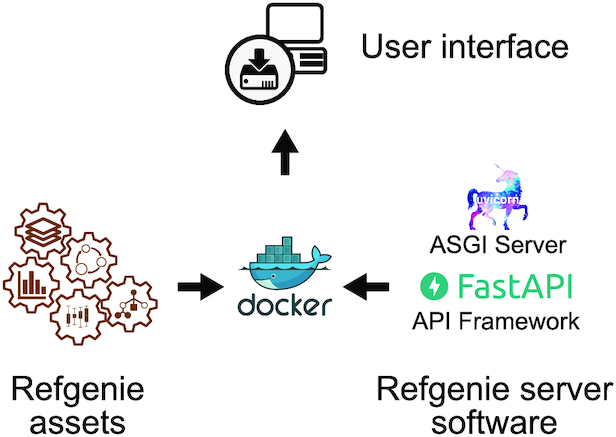
Server software stack. Archived refgenie assets are mounted into a Docker container, along with the refgenie server software, which is built using FastAPI and uvicorn. The container can then be accessed via the web and API user interfaces.

This server software leverages cutting-edge web technology to provide high-concurrency service with minimal compute resources (Fig. 3). We built refgenieserver on top of the FastAPI Python framework, which is a high-performance web framework for building APIs. FastAPI automatically produces an API that complies with OpenAPI 3.0 standards, which will allow other tools to discover and automatically use the API. It also includes a self-documenting test interface so that users can see and test the available API end points. Refgenie leverages the Starlette development toolkit and the uvicorn server to make use of the high-performance Asynchronous Server Gateway Interface (ASGI) specification, which provides asynchronous access to refgenieserver.

Refgenieserver is containerized and available on dockerhub, so an interested user could start a server with a single line of code:

docker run --rm -p 80:80 \

-v genomes_folder:/genomes rgsimage \

refgenieserver serve -c /genomes/config.yaml

By mounting a refgenie “genomes" folder into this container, users get a fully functioning web interface and RESTful API.

### Refgenconf package for genome configuration

Refgenie organizes assets by genome in the configuration file, which is both computer-readable and human-readable. In practice, users will not need to interact with this file at all because refgenie will handle both reading and writing the file. However, users may edit the file if they need a more complicated structure (such as storing assets on different file systems or adding assets manually). Together with the refgenie software, this simple file makes the concept of reference genome assets completely portable. Full documentation for the configuration file format can be found at http://refgenie.databio.org.

The configuration package, refgenconf, simply provides functions and data types to read and write items listed in the genome configuration file. Under the hood, the refgenie CLI itself uses refgenconf to interact with the genome configuration and assets on disk. The server software also relies on it to read, archive, and serve assets. The refgenconf package also provides the starting point for any third-party Python developers by providing a fully functional Python API for interacting with refgenie assets. For example, we use refgenconf in Python pipelines that we develop to make them aware of the genome assets available in a given computing environment. Using this approach, a pipeline need only be provided with an assembly key, such as “hg38," and it can use refgenconfto locate the correct path to any genome-related asset necessary for the pipeline. This simplifies the process of configuring pipelines and allows refgenie to be used both by humans and computers.

### The Refgenomes database

We designed the server software so that anyone could easily run a custom server instance. We have also deployed our own instance of refgenieserver at refgenomes.databio.org, where we host pre-built genome assets. Like any instance of refgenieserver, our refgenomes database provides both a web interface and a RESTful API to access individual assets that we have made available. Users may explore and download archived indexes from the web interface or develop tools that programmatically query the API.

The web interface provides a graphical listing of available genomes and assets, allowing users to browse the site and download individual assets manually. In addition, refgenieserver provides API end points to serve lists of available genomes and assets, as well as metadata for the individual assets, including unique digests for file integrity, file sizes, and archive content information. Furthermore, the server provides end points to download each asset individually. End points include the following: /genomes retrieves a list of available genomes; /assets retrieves a list of all available assets;/{genome}/assets/ retrieves a list of assets for a given genome; and /{genome}/assets/{asset}/archive retrieves the tarball for the specified asset. Complete documentation is available at refgenomes.databio.org. Because it provides a standard OpenAPI-compliant RESTful API, our server will be useful not just for our refgenie CLI but for other tools that would benefit from automated access to reference assembly assets and indexes.

Ourrefgenieserver instance runs within DC/OS as a containerized application. The server application makes genome assets available through a web application connected directly to a remote filesystem, with no additional database or infrastructure requirements. Integration and deployment is automated using GitHub, Travis-CI, Docker Hub, and a custom deployment technique made simple in DC/OS. Changes committed in code are generally deployed to development or production services within 1–3 minutes.

### Genome provenance

One challenge with genome assembly assets is name mismatches that lead to analysis conflicts. Because refgenie identifiers are human-readable and user-controlled, refgenie cannot rely on them to uniquely identify assets. Furthermore, refgenie assets may be either built or pulled from different servers, exacerbating the issue. This is an active area of research, with several approaches under development related to this problem, such as the NCBI Assembly database [4], the refget protocol for sequence identifiers [19], and tximeta checksums for RNA-sequencing data [20]. Refgenie currently provides 2 resources to confirm the identity of pulled and built assets: first, a unique digest for each asset, and second, a building log file. Refgenie makes unique asset digests available via both web interface and API, allowing users to distinguish between 2 assets with the same names but different digests. Furthermore, because building refgenie assets is scripted, it is possible to trace any asset back to its inputs. Refgenieserver provides API points to retrieve either the raw recipe (/v2/asset/{genome}/{asset}/recipe) or the actual log file(/v2/asset/{genome}/{asset}/log) for any asset available on the server. For built assets, the build command automatically produces a log file that records the input files, software versions, and final digests for any locally built assets. These resources make it possible for users to uniquely identify and trace the provenance of assets they either build or pull.

## Comparison with Existing Tools

**Figure 4:**
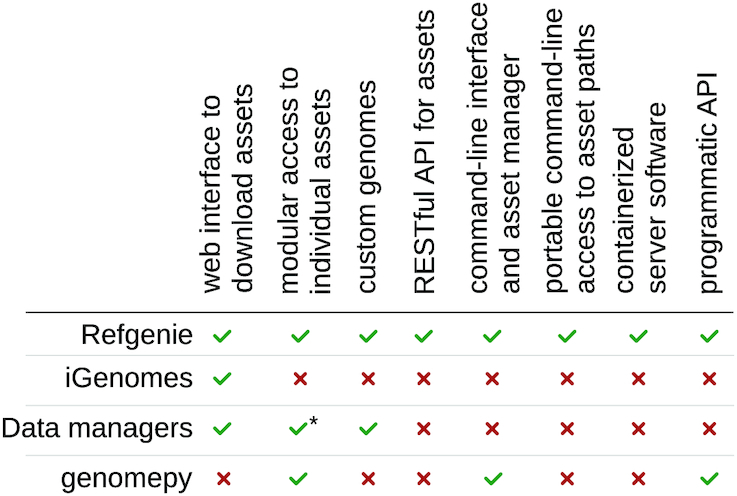
Feature comparison. iGenomes, Galaxy Data managers, and genomepy solve some problems of standardized reference genome assets but lack the interactive features of refgenie. *Data managers' assets can be accessed individually but not outside of the Galaxy user interface.

A few existing tools approach these problems as well. The most similar projects are Illumina’s iGenomes and Galaxy Data Managers accompanied by Galaxy Tool Shed [21,22], both of which offer only a small part of what refgenie does (Fig. 4). iGenomes provides a single archive download of a standardized folder structure with pre-built assets for pre-defined genomes. The Data Managers facilitate building of assets; they are tightly coupled to the larger Galaxy infrastructure, while Refgenie’s modular design allows for simple implementation in diverse environments. The genomepy tool provides a unified command line interface and Python API to download genome sequences from multiple sources but does not accommodate custom genomes and has no remote API or component for downloading indexes [23]. Some of refgenie’s utility is also satisfied by individual tool websites that provide individual asset downloads (e.g., bowtie2 indexes), but these provide no shared structure or unified interface for access.

Refgenie provides a full-service manager that unifies and transcends all of these available tools. Refgenie solves a series of related problems all in 1 convenient package. It provides a unified web interface for all assets, plus programmatic access to modular individual assets via a RESTful API for metadata and assets. Refgenie also provides the ability to build assets for custom genomes with a uniform interface that integrates seamlessly with downloaded assets. Refgenie is unique in providing a local asset manager that makes locating assets portable, simplifying building pipelines that use these assets. It is also the only easily deployable, independent, containerized server application and Python API for reference genome assets. Thus, no existing software can solve these problems specific to genome-related data resources.

## Conclusions and Future Directions

Reference genomes, indexes, annotations, and other genome assets are integral to sequencing analysis projects, and these genome-associated data resources are growing rapidly [11]. Refgenie provides a full-service management system that includes a convenient method for downloading, building, sharing, and using these resources. Refgenieserver is among a growing number of API-oriented projects in the life sciences [5,24,25]. Refgenie will simplify management of reference assembly assets for users and groups, facilitating data sharing and software interoperability [26].

Several new features under development will make refgenie even more useful. Currently, refgenie is completely flexible with respect to genomes, but it is less flexible with respect to assets because only pre-scripted assets can be built. A more flexible approach would allow refgenie to accept custom recipes, allowing users to add new asset types. Future development will address the challenges of sharing recipes, provenance, and trust for flexible assets. We are also improving the way refgenie records and uses identifiers and relationships among assets. For instance, by recording more detailed information about what an asset contains and how it was generated, we open the possibility of delineating more fine-grained compatibilities. For instance, while 2 indexes would be compatible only if derived from the same set of sequences, 2 annotation files could be compatible on different sequences that shared a coordinate structure. Finally, we anticipate that future development will extend refgenie to be able to accommodate ontology annotation for assets and genomes. Together, these improvements will enable more robust discovery of assets and genomes, as well as the relationships among them.

## Availability of Source Code and Requirements

Project name: Refgenie

Project home page: http://refgenie.databio.org

Operating system: Platform independent

Programming language: Python

Other requirements: Varies by use case

License: BSD-2

RRID:SCR_017574

biotools ID: Refgenie

An archival copy of the code is available via the *GigaScience* database, GigaDB [27].

## Abbreviations

API: Application Programming Interface; ASGI: Asynchronous Server Gateway Interface; CLI: command line interface; DC/OS: distributed cloud operating system; NCBI: National Center for Biotechnology Information: REST: representational state transfer; URL: universal resource locator.

## Competing interests

The authors declare that they have no competing interests.

## Authors' contributions

NCS conceived the study. MS, VPR, and NS wrote the software with contributions from JPS and NEM. All authors contributed to and approved the manuscript.

## Supplementary Material

giz149_GIGA-D-19-00289_Original_Submission

giz149_GIGA-D-19-00289_Revision_1

giz149_GIGA-D-19-00289_Revision_2

giz149_Response_to_Reviewer_Comments_Original_Submission

giz149_Response_to_Reviewer_Comments_Revision_1

giz149_Reviewer_1_Report_Original_Submission Andrew Yates -- 8/14/2019 Reviewed

giz149_Reviewer_2_Report_Original_Submission Katherine James -- 8/26/2019 Reviewed

giz149_Reviewer_3_Report_Original_Submission Bernie Pope, Ph.D. -- 8/29/2019 Reviewed

giz149_Reviewer_3_Report_Revision_1 Bernie Pope, Ph.D. -- 10/24/2019 Reviewed

giz149_Reviewer_4_Report_Original_Submission Christophe Dessimoz -- 9/4/2019 Reviewed
