# Supplementary material for: Refgenie: a reference genome resource manager
Source: Gigascience. 2020 Jan 29;9(2):giz149. doi: 10.1093/gigascience/giz149 (PMC6988606; doi:10.1093/gigascience/giz149)
Supplement: giz149_GIGA-D-19-00289_Original_Submission [file giz149_giga-d-19-00289_original_submission.pdf]

GigaScience

Refgenie: a reference genome resource manager

--Manuscript Draft--

**Manuscript Number:**	GIGA-D-19-00289
**Full Title:**	Refgenie: a reference genome resource manager
**Article Type:**	Technical Note
**Funding Information:**	National Institute of General Medical Sciences (1R35GM128636-01) — Dr. Nathan C. Sheffield
**Abstract:**	Reference genome assemblies are essential for high-throughput sequencing analysis projects. Typically, genome assemblies are stored on disk alongside related resources; for example, many sequence aligners require the assembly to be *indexed*. The resulting indexes are broadly applicable for downstream analysis, so it makes sense to share them. However, there is no simple tool to do this. To this end, we introduce refgenie, a reference genome assembly asset manager. Refgenie makes it easier to organize, retrieve, and share genome analysis resources. In addition to genome indexes, refgenie can manage any files related to reference genomes, including sequences and annotation files. Refgenie includes a command-line interface and a server application that provides a RESTful API, so it is useful for both tool development and analysis. **Availability:** https://refgenie.databio.org
**Corresponding Author:**	Nathan C. Sheffield<br><br>UNITED STATES
**Corresponding Author Secondary Information:**	
**Corresponding Author's Institution:**	
**Corresponding Author's Secondary Institution:**	
**First Author:**	Michał Stolarczyk
**First Author Secondary Information:**	
**Order of Authors:**	Michał Stolarczyk<br>Vincent Reuter<br>Nathan C. Sheffield<br>Neal Magee
**Order of Authors Secondary Information:**	
**Additional Information:**	
**Question**	**Response**
Are you submitting this manuscript to a special series or article collection?	No
**Experimental design and statistics**<br><br>Full details of the experimental design and statistical methods used should be given in the Methods section, as detailed in our Minimum Standards Reporting Checklist. Information essential to interpreting the	No

Powered by Editorial Manager® and ProduXion Manager® from Aries Systems Corporation

data presented should be made available in the figure legends.<br><br>Have you included all the information requested in your manuscript?	
If not, please give reasons for any omissions below.<br><br>as follow-up to "**Experimental design and statistics**<br><br>Full details of the experimental design and statistical methods used should be given in the Methods section, as detailed in our Minimum Standards Reporting Checklist. Information essential to interpreting the data presented should be made available in the figure legends.<br><br>Have you included all the information requested in your manuscript?<br><br>"	No experiments performed.
**Resources**<br><br>A description of all resources used, including antibodies, cell lines, animals and software tools, with enough information to allow them to be uniquely identified, should be included in the Methods section. Authors are strongly encouraged to cite Research Resource Identifiers (RRIDs) for antibodies, model organisms and tools, where possible.<br><br>Have you included the information requested as detailed in our Minimum Standards Reporting Checklist?	Yes
**Availability of data and materials**<br><br>All datasets and code on which the conclusions of the paper rely must be	Yes

either included in your submission or deposited in publicly available repositories (where available and ethically appropriate), referencing such data using a unique identifier in the references and in the "Availability of Data and Materials" section of your manuscript.<br><br>Have you have met the above requirement as detailed in our Minimum Standards Reporting Checklist?	

Powered by Editorial Manager® and ProduXion Manager® from Aries Systems Corporation

Manuscript Click here to download Manuscript cover_letter.pdf

UNIVERSITY of VIRGINIA
SCHOOL of MEDICINE

Center for Public Health Genomics

P.O. Box 800717
Charlottesville, VA 22908-0717

P 434-982-3228
F 434-982-1815
med.virginia.edu/cphg

August 8, 2019

Dear Editor,

Please consider our attached manuscript describing our software, *refgenie*. Refgenie is full-service reference genome manager that organizes storage, access, and transfer of reference genomes. It provides command-line and python interfaces to download pre-built reference genome “assets” like indexes used by bioinformatics tools. It can also build assets for custom genome assemblies. Refgenie provides programmatic access to a standard genome folder structure, so software can swap from one genome to another.

Refgenie is the first system to provide both automated build and download. This is a new approach to this very fundamental problem in genomics. The most common approach currently is to just place resources like genome indexes on a web server for manual download. This, for example, is done by Illumina’s iGenomes project, which distributes pre-built resources. Refgenie re-envisions this process, providing a modular API that allows users to download individual assets. More important, refgenie also allows users to build reference genome resources for arbitrary genomes locally, so you don’t have to rely on a server component at all. This is not currently possible with any existing system. As a result, we are unable to put in a meaningful comparison to existing software, because refgenie is the first in its class. We have added a section detailing how refgenie compares to the iGenomes approach, but the comparison is quite one-sided, as iGenomes is not even software at all, so it obviously lacks all of the features of refgenie.

Since placing a preprint on biorxiv, Refgenie has already achieved substantial notoriety and is in the top 5% of research articles posted to biorxiv. We believe this manuscript to be commensurate with the expectations of your reviewers and readers. We believe *GigaScience* is the right journal for this work because of its breadth and utility across many biological disciplines. Refgenie will be of interest to computational researchers from a methods perspective, and definitely to genomics researchers as a resource – but will also be useful to anyone who even casually uses genome-related resources or data, which is common across biology. As such, we feel that a general audience would be ideal for this manuscript.

Sincerely,

Nathan Sheffield

Nathan Sheffield, PhD, on behalf of all co-authors
Assistant Professor, Center for Public Health Genomics, University of Virginia
www.databio.org
434-924-8278

Click here to download Personal Cover manuscript.pdf

RESEARCH ARTICLE

Refgenie: a reference genome resource manager

Michał Stolarczyk[1, *], Vincent P. Reuter[1, *], Neal E. Magee[5], and Nathan C. Sheffield[1,2,3,4,✉]

[1]Center for Public Health Genomics, University of Virginia
[2]Department of Public Health Sciences, University of Virginia
[3]Department of Biomedical Engineering, University of Virginia
[4]Department of Biochemistry and Molecular Genetics, University of Virginia
[5]Research Computing, University of Virginia
[*]Contributed equally
✉ Correspondence: nsheffield@virginia.edu

Reference genome assemblies are essential for high-throughput sequencing analysis projects. Typically, genome assemblies are stored on disk alongside related resources; for example, many sequence aligners require the assembly to be *indexed*. The resulting indexes are broadly applicable for downstream analysis, so it makes sense to share them. However, there is no simple tool to do this. To this end, we introduce refgenie, a reference genome assembly asset manager. Refgenie makes it easier to organize, retrieve, and share genome analysis resources. In addition to genome indexes, refgenie can manage any files related to reference genomes, including sequences and annotation files. Refgenie includes a command-line interface and a server application that provides a RESTful API, so it is useful for both tool development and analysis.
Availability: `https://refgenie.databio.org`

Background

Enormous effort goes into assembling and curating reference genomes[1–5]. These reference assemblies provide a common representation for comparing results and they form the basis for a wide range of downstream tools for sequence alignment and annotation. Many tools that rely on reference assemblies will produce independent resources that accompany an assembly. For instance, many aligners must *hash* the genome, creating *indexes* that are used to improve alignment performance[6–9].

Analytical pipelines typically rely on these aligners and their indexes for the initial steps of a data analysis. These assembly resources are typically shared among many pipelines, so it's common for a research group to organize them in a central folder to prevent duplication. In addition to saving disk space, centralization simplifies sharing software that uses a reference assembly because software can be written around a standard folder structure. However, this does not solve the problem of sharing genomic resources *between* research groups. Because each group may use a different strategy to identify shared genome resources, sharing tools across groups may require modifying them.

One solution to this problem is to have a web-accessible server where standard, organized reference assemblies are available for download. Indeed, this is exactly the goal of Illumina's *iGenomes* project, which provides "a collection of reference sequences and annotation files for commonly analyzed organisms"[10]. The iGenomes project has become a popular source of genome assets and has greatly simplified sharing analysis tools among research environments. However, this approach suffers from some fundamental drawbacks and leaves several challenges unsolved. First, the individual assets can only be downloaded in bulk, but what if a particular use case requires only a small subset of resources in a package? More important, building the resources is not scripted, so if the repository excludes a reference or resource of interest, there is no programmatic way to fill the gap. In these scenarios, users must manually build and organize genome assets individually, forfeiting the strength of standardization among groups.

To improve the ability to share interoperable reference genome assets, we have developed *refgenie*, which enables a more modular, customizable, and user-controlled approach to managing reference assembly resources. Like iGenomes, refgenie standardizes reference genome asset organization so software can be built around that organization. But unlike iGenomes, refgenie also automates the *building* of genome assets, so that an identical representation can be produced for any genome assembly. Furthermore, refgenie allows programmatic access to individual resources both remote and local, making it suitable for the next generation of self-contained pipelines.

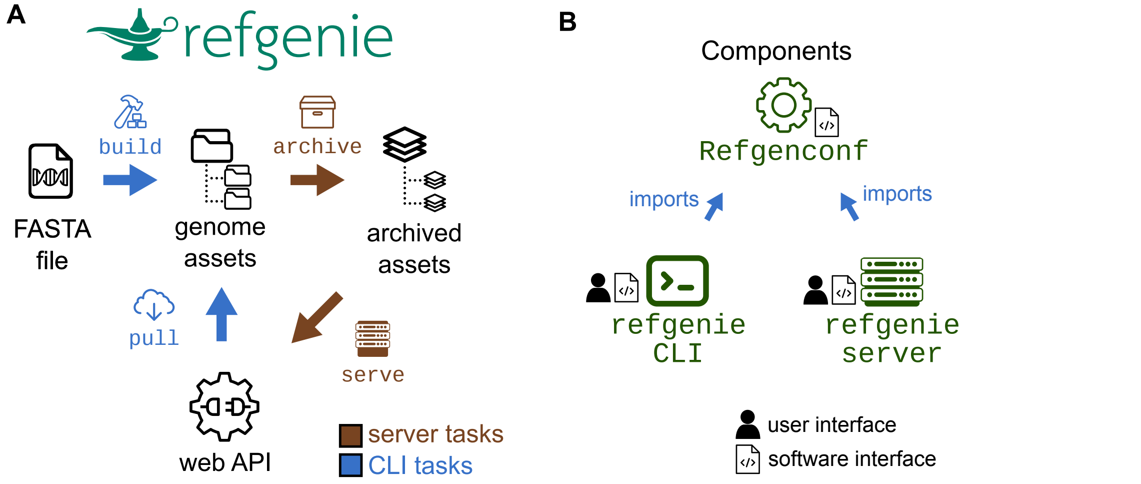

*Fig. 1: **Refgenie concept and software organization**. A: Refgenie provides the ability to either build or pull assets. B: Refgenie is tripartite, made up of a `conf` utility, a command-line interface (CLI), and a server package. The configuration package is intended for programmatic use, and is used by the CLI and server packages. Users and software use refgenie via the CLI or server (web API).*

Refgenie can organize any files that can be assigned to a particular reference genome assembly, which could include not only genome indexes, but other resource types like genome sequences and annotations[11–13].

Refgenie manages genome-related resources flexibly. It can handle any asset type, from annotations to indexes. It provides individual, pre-built asset downloads from a server and allows scripted building for custom inputs. Refgenie thus solves a major hurdle in biological data analysis.

```
genome_folder: /genomes/path
genome_server: http://...
config_version: 0.2
genomes:
  hg38:
    genome_description: ...
    assets:
      bowtie2_index:
        path: bowtie2_index
        asset_description: ...
      hisat2_index:
        path: hisat2_index
        asset_description: ...
```

*Fig.2: **Genome config file.** Refgenie reads and writes a genome configuration file in YAML format to keep track of available local assets.*

Results and discussion

Refgenie is the first full-service *reference genome asset manager*. Refgenie provides two ways to obtain genome assets: *pull*, and *build* (Fig.1A). For common assets, *pulling* a pre-built version obviates the need to install and run specialized software to build a particular asset. It also makes it easier to satisfy prerequisites programmatically for pipelines and other software. However, remote-hosted assets are only practical for common genomes and assets, so for uncommon assets or on unconnected computers, users may instead *build* assets, which creates the same standard output for custom genomes. By providing both *build* and *pull*, refgenie facilitates asset organization both within and between research groups, increasing interoperability of tools that rely on genome resources.

The `refgenie` software suite consists of three components: 1) a command-line interface (CLI), 2) a server, and 3) a configuration package that supports them both (Fig.1B). Each of these relies on a local `YAML` file called the *genome configuration file* (Fig. 2), which refgenie uses to keep track of metadata, such as local file paths.

Genome configuration and asset organization

Refgenie organizes genome assembly resources into *assets*, each of which represents one or more files. You can think of a genome asset as a folder of related files tied to a particular genome assembly. For example, an asset could be an index for a particular tool, or a group of annotation files. Refgenie organizes such assets by genome in the configuration file, which is both computer-readable and human-readable. In practice, users will not need to interact with this file at all, as refgenie will handle both reading and writing the file. However, users may edit the file if they need a more complicated structure (such as storing assets on different file systems, or adding assets manually). Together with the refgenie software, this simple file makes the concept of reference genome assets completely portable. Full documentation for the configuration file format can be found at refgenie.databio.org.

Refgenconf configuration package

The configuration package, `refgenconf`, simply provides functions and data types to read and write items listed in the genome configuration file.

Under the hood, the refgenie CLI itself uses `refgenconf` to interact with the genome configuration and assets on disk. The server software also relies on it to read, archive, and serve assets. The `refgenconf` package also provides the starting point for any third-party python developers by providing a fully functional python application programming interface (API) for interacting with refgenie assets. For example, we use `refgenconf` in python pipelines we develop to make them aware of the genome assets available in a given computing environment. Using this approach, a pipeline need only be provided with an assembly key, like 'hg38', and it can use `refgenconf` to locate the correct path to any genome-related asset necessary for the pipeline. This simplifies the process of configuring pipelines and allows `refgenie` to be used both by humans and computers.

Refgenie command-line interface

The workhorse of refgenie is the command-line interface (CLI); it is how users will typically interact with genome assets. Its implementation as a command-line tool not only makes it useful for general purpose exploration and access, but also allows it to be integrated into existing workflows that require access to genome assets from the shell. The CLI can be installed with `pip install refgenie` and invoked by calling `refgenie`. The refgenie CLI provides 5 functions for interacting with local genome assets:

- `refgenie init` initializes an empty genome configuration file
- `refgenie list` summarizes the genome configuration file, listing local genomes and assets
- `refgenie seek` provides the file path to a given asset
- `refgenie add` adds an already-built local asset
- `refgenie build` builds a new asset

The `init`, `list`, `seek`, and `add` functions follow directly from the configuration file format. They essentially allow a user to easily explore and access file paths to available assets. The `build` function allows a user to *build* assets for any FASTA file, which is a more flexible system than alternative approaches that provide only downloadable assets. Refgenie has built-in capability to build a selection of different common genome assets (Fig.3). In addition to functions on local assets, the refgenie CLI also contains additional commands that can interact with remote assets: *pull* and *listr*:

- `refgenie listr` lists available remote genomes and assets
- `refgenie pull` downloads a remote asset

With these commands, refgenie makes downloading a standard index for a user as simple as a few lines of code in a shell. For example, a new user can initialize refgenie and then download the bowtie2 indexes for the hg38 reference with these lines of code:

```
pip install --user refgenie
refgenie init -c conf.yaml
refgenie pull -c conf.yaml -g hg38 -a ASSET
```

Where `ASSET` is a unique key defining the asset of interest (*e.g.*, `bowtie2_index`). Once the asset has been pulled (or built), the user can retrieve the path to it with `refgenie seek`:

```
refgenie seek -c config.yaml -g hg38 -a ASSET
```

This command returns the file path to the specified asset for the specified genome. This command is now portable, eliminating the need to hard-code paths, or pass them as arguments, in a pipeline or other software that requires access to genome assembly assets.

Refgenie server

The `listr` and `pull` functions require that the CLI interact with a server. The CLI uses a configurable URL to retrieve a remote archived tarball. After retrieving the tarball, the CLI will unpack it into the appropriate folder location and update the configuration file to provide access to its path via `refgenie seek`.

To support this remote function, we have developed a containerized, portable, open-source companion application called `refgenieserver`. Many users of refgenie will not have to be aware of the server application; however, interested users can use refgenie server to host their own genome asset server. For example, a tool developer may wish to simplify use by hosting indexes for common reference assemblies.

Running the refgenie server is simple for users who are already familiar with refgenie. It reads the same genome configuration file format as the CLI (indeed, it uses the `refgenconf` package described earlier in the same way). In fact, refgenie server operates on the same genome config file and asset folders that that

asset name	genome	asset size	archive size	build time	peak memory
fasta	hg38	2.9 GB	0.8 GB	0:51:43	0 GB
bowtie2_index	hg38	3.9 GB	3.5 GB	0:57:29	5.6 GB
hisat2_index	hg38	4.2 GB	3.9 GB	0:36:22	5.5 GB
bismark_bt1_index	hg38	13.6 GB	7.5 GB	1:10:24	10.8 GB
bismark_bt2_index	hg38	13.6 GB	7.5GB	2:17:22	10.8 GB
bwa_index	hg38	2.9 GB	3.2 GB	0:51:02	4.7 GB
star_index	hg38	26.9 GB	24.3GB	1:51:11	35.8 GB
kallisto_index	hg38_cdna	2.2 GB	1.6 GB	0:04:30	3.8 GB
salmon_index	hg38_cdna	3.1 GB	2.6GB	0:03:04	5.3 GB

*Fig.3: **Assets available for build**. Table listing assets that can currently be built with `refgenie build`, along with statistics for size, build time, and memory high water mark. Assets were built for the human genome using a single core. Times and memory are representative values from a single run. These assets are produced by various tools[8,9,14–17] and are available to be built for any arbitrary genome input.*

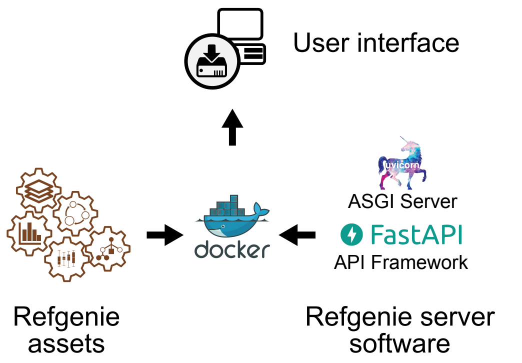

*Fig.4: **Server software stack**. Archived refgenie assets are mounted into a Docker container, along with the refgenie server software, which is built using FastAPI and uvicorn. The container can then be accessed via the web and API user interfaces.*

refgenie itself builds or downloads. The server software comes with an `archive` command that prepares a refgenie genome folder for serving. It compresses each asset into an individual tarball. This simple system makes it easy for users to run a server using their refgenie assets.

This server software leverages cutting-edge web technology to provide high-concurrency service with minimal compute resources (Fig. 4). We built refgenie server on top of the FastAPI python framework, which is a high performance web framework for building APIs. FastAPI automatically produces an API that complies with OpenAPI 3.0 standards, which will allow other tools to discover and automatically use the API. It also includes a self-documenting test interface so that users can see and test the available API endpoints. Refgenie leverages the Starlette development toolkit and the `uvicorn` server to make use of the lightning-fast Asynchronous Server Gateway Interface (ASGI) specification, which provides asynchronous access to refgenie server.

Refgenie server is containerized and available on dockerhub, so that an interested user could run a server with a single line of code:

```
docker run --rm -p 80:80 \
  -v genomes_folder:/genomes rgimage \
   refgenie -c /genomes/config.yaml serve
```

By mounting a refgenie 'genomes' folder into this container, users get a fully functioning web interface and RESTful API.

The Refgenomes database

We designed the server software so that anyone could easily run a custom server instance. We have also deployed our own instance of `refgenieserver` at refgenomes.databio.org, where we host pre-built genome assets. Like any instance of `refgenieserver`, our refgenomes database provides both a web interface and a RESTful API to access individual assets we have made available. Users may explore and download archived indexes from the web interface or develop tools that programmatically query the API.

The web interface provides a graphical listing of available genomes and assets, allowing users to browse the site and download individual assets manually. In addition, refgenieserver provides API endpoints to serve lists of available genomes and assets, as well as metadata for the individual assets, including checksums for file integrity, file sizes, and archive content information. Furthermore, the server provides endpoints to download each asset individually. Endpoints include the following: `/genomes` retrieves a list of available genomes; `/assets` retrieves a list of all available assets; `/{genome}/assets/` retrieves a list of assets for a given genome; and `/{genome}/assets/{asset}/archive` retrieves the tarball for the specified asset. Complete documentation is available at refgenomes.databio.org. Because it provides a standard OpenAPI-compliant RESTful API, our server will be useful not just for our refgenie CLI, but for other tools that would benefit

	web interface to download assets	modular access to individual assets	custom genomes	RESTful API for assets	command-line interface and asset manager	containerized server software	python API
Refgenie	✓	✓	✓	✓	✓	✓	✓
iGenomes	✓	✗	✗	✗	✗	✗	✗

*Fig.5: **Feature comparison**. iGenomes also solves the problem of standardized reference genome assets, but it is a simple archive download that lacks the interactive features of refgenie.*

from automated access to reference assembly assets and indexes.

Our `refgenieserver` instance runs within DC/OS as a containerized application managed by Marathon. Marathon deploys each application stack separately, monitors individual container health, and connects them to remote NFS storage and HTTP load balancers as appropriate. Marathon also has the ability to autoscale cluster deployments. The refgenie application makes genome assets available through a web application connected directly to a remote filesystem, with no additional database or infrastructure requirements. Integration and deployment of frequently updated components is automated using GitHub, Travis-CI, Docker Hub, and a custom deployment technique made simple in DC/OS. Changes committed in code are generally deployed to development or production services within 1-3 minutes.

Comparison to existing tools

Refgenie fills a niche for which, to our knowledge, there is no other competing software. The most similar project is Illumina's *iGenomes*, which satisfies only a small part of what refgenie accomplishes (Fig. 5). iGenomes provides a single archive download of a standardized folder structure with pre-build assets for pre-defined genomes. Refgenie provides a full-service manager that allows modular API-based access either via web or command-line interface, provides a python interface. Furthermore, refgenie manages local paths to assets, simplifying building pipelines that use these assets. No existing software can solve these problems specific to genome-related data resources.

Conclusions

Reference genomes, indexes, annotations, and other genome assets are integral to sequencing analysis projects, and these genome-associated data resources are growing rapidly[11]. Refgenie provides a full-service management system that includes a convenient method for downloading, building, sharing, and using these resources. Refgenieserver is among a growing number of API-oriented projects in the life sciences[5,18,19]. Refgenie will simplify management of reference assembly assets for users and groups, facilitating data sharing and software interoperability[20].

License & availability

Refgenie, refgenieserver, and refgenconf python packages are all BSD2-licensed. Source code and documentation can be found at refgenie.databio.org.

References

1. Harrow, J. *et al.* GENCODE: The reference human genome annotation for the ENCODE project. *Genome Research* **22,** 1760–1774 (2012).

2. Pruitt, K. D., Tatusova, T., Brown, G. R. & Maglott, D. R. NCBI reference sequences (RefSeq): Current status, new features and genome annotation policy. *Nucleic Acids Research* **40,** D130–D135 (2011).

3. Church, D. M. *et al.* Modernizing reference genome assemblies. *PLoS Biology* **9,** e1001091 (2011).

4. Kitts, P. A. *et al.* Assembly: A resource for assembled genomes at NCBI. *Nucleic Acids Research* **44,** D73–D80 (2015).

5. Ruffier, M. *et al.* Ensembl core software resources: Storage and programmatic access for DNA sequence and genome annotation. *Database* **2017,** (2017).

6. Sadakane, K. & Shibuya, T. Indexing huge genome sequences for solving various problems. *Genome Informatics* **12,** 175–183 (2001).

7. Hon, W.-K., Sadakane, K. & Sung, W.-K. Breaking a time-and-space barrier in constructing full-text indices. *SIAM Journal on Computing* **38,** 2162–2178 (2009).

8. Li, H. & Durbin, R. Fast and accurate short read alignment with burrows-wheeler transform. *Bioinformatics* **25,** 1754–60 (2009).

9. Langmead, B. & Salzberg, S. L. Fast gapped-read alignment with bowtie 2. *Nat. Methods* **9,** 357–359 (2012).

10. Illumina. IGenomes. Ready-to-use reference sequences and annotations. *support.illumina.com* (2019).

11. Richa Agarwala *et al.* Database resources of the national center for biotechnology information. *Nucleic Acids Research* **46,** D8–D13 (2018).

12. Zerbino, D. R., Wilder, S. P., Johnson, N., Juettemann, T. & Flicek, P. R. The Ensembl Regulatory Build. *Genome Biology* **16,** (2015).

13. Sheffield, N. C. & Bock, C. LOLA: Enrichment analysis for genomic region sets and regulatory elements in R and bioconductor. *Bioinformatics* **32,** 587–589 (2016).

14. Krueger, F. & Andrews, S. R. Bismark: A flexible aligner and methylation caller for bisulfite-seq applications. *Bioinformatics* **27,** 1571–1572 (2011).

15. Bray, N. L., Pimentel, H., Melsted, P. & Pachter, L. Near-optimal probabilistic RNA-seq quantification. *Nature Biotechnology* **34,** 525–527 (2016).

16. Kim, D., Langmead, B. & Salzberg, S. L. HISAT: A fast spliced aligner with low memory requirements. *Nature Methods* **12,** 357–360 (2015).

17. Dobin, A. *et al.* STAR: Ultrafast universal RNA-seq aligner. *Bioinformatics* **29,** 15–21 (2012).

18. Yates, A. *et al.* The ensembl REST API: Ensembl data for any language. *Bioinformatics* **31,** 143–145 (2014).

19. Tarkowska, A. *et al.* Eleven quick tips to build a usable REST API for life sciences. *PLOS Computational Biology* **14,** e1006542 (2018).

20. Wilkinson, M. D. *et al.* The FAIR guiding principles for scientific data management and stewardship. *Sci. Data* **3,** 160018 (2016).
