# Supplementary material for: Refgenie: a reference genome resource manager
Source: Gigascience. 2020 Jan 29;9(2):giz149. doi: 10.1093/gigascience/giz149 (PMC6988606; doi:10.1093/gigascience/giz149)
Supplement: giz149_GIGA-D-19-00289_Revision_1 [file giz149_giga-d-19-00289_revision_1.pdf]

GigaScience

Refgenie: a reference genome resource manager

--Manuscript Draft--

**Manuscript Number:**	GIGA-D-19-00289R1
**Full Title:**	Refgenie: a reference genome resource manager
**Article Type:**	Technical Note
**Funding Information:**	National Institute of General Medical Sciences (1R35GM128636-01) — Dr. Nathan C. Sheffield
**Abstract:**	Reference genome assemblies are essential for high-throughput sequencing analysis projects. Typically, genome assemblies are stored on disk alongside related resources; for example, many sequence aligners require the assembly to be *indexed*. The resulting indexes are broadly applicable for downstream analysis, so it makes sense to share them. However, there is no simple tool to do this. To this end, we introduce refgenie, a reference genome assembly asset manager. Refgenie makes it easier to organize, retrieve, and share genome analysis resources. In addition to genome indexes, refgenie can manage any files related to reference genomes, including sequences and annotation files. Refgenie includes a command-line interface and a server application that provides a RESTful API, so it is useful for both tool development and analysis.<br><br>**Availability:** https://refgenie.databio.org
**Corresponding Author:**	Nathan C. Sheffield<br><br>UNITED STATES
**Corresponding Author Secondary Information:**	
**Corresponding Author's Institution:**	
**Corresponding Author's Secondary Institution:**	
**First Author:**	Michał Stolarczyk
**First Author Secondary Information:**	
**Order of Authors:**	Michał Stolarczyk
	Vincent Reuter
	Jason P. Smith
	Neal Magee
	Nathan C. Sheffield
**Order of Authors Secondary Information:**	
**Response to Reviewers:**	Response to review is included as a PDF cover letter for ease of review.
**Additional Information:**	
**Question**	**Response**
Are you submitting this manuscript to a special series or article collection?	No
**Experimental design and statistics**<br><br>Full details of the experimental design and	No

Powered by Editorial Manager® and ProduXion Manager® from Aries Systems Corporation

statistical methods used should be given in the Methods section, as detailed in our Minimum Standards Reporting Checklist. Information essential to interpreting the data presented should be made available in the figure legends.<br><br>Have you included all the information requested in your manuscript?	
If not, please give reasons for any omissions below.<br><br>as follow-up to "**Experimental design and statistics**<br><br>Full details of the experimental design and statistical methods used should be given in the Methods section, as detailed in our Minimum Standards Reporting Checklist. Information essential to interpreting the data presented should be made available in the figure legends.<br><br>Have you included all the information requested in your manuscript?<br><br>"	No experiments performed.
**Resources**<br><br>A description of all resources used, including antibodies, cell lines, animals and software tools, with enough information to allow them to be uniquely identified, should be included in the Methods section. Authors are strongly encouraged to cite Research Resource Identifiers (RRIDs) for antibodies, model organisms and tools, where possible.<br><br>Have you included the information requested as detailed in our Minimum Standards Reporting Checklist?	Yes
**Availability of data and materials**	Yes

All datasets and code on which the conclusions of the paper rely must be either included in your submission or deposited in publicly available repositories (where available and ethically appropriate), referencing such data using a unique identifier in the references and in the “Availability of Data and Materials” section of your manuscript.<br><br>Have you have met the above requirement as detailed in our Minimum Standards Reporting Checklist?	

Powered by Editorial Manager® and ProduXion Manager® from Aries Systems Corporation

Manuscript
Click here to access/download;Manuscript;manuscript.pdf
Click here to view linked References

RESEARCH ARTICLE

Refgenie: a reference genome resource manager

Michał Stolarczyk[1,*], Vincent P. Reuter[1,*], Jason P. Smith[1,4], Neal E. Magee[5], and Nathan C. Sheffield[1,2,3,4,✉]

[1]Center for Public Health Genomics, University of Virginia
[2]Department of Public Health Sciences, University of Virginia
[3]Department of Biomedical Engineering, University of Virginia
[4]Department of Biochemistry and Molecular Genetics, University of Virginia
[5]Research Computing, University of Virginia
[*]Contributed equally
✉ Correspondence: nsheffield@virginia.edu

Reference genome assemblies are essential for high-throughput sequencing analysis projects. Typically, genome assemblies are stored on disk alongside related resources; for example, many sequence aligners require the assembly to be *indexed*. The resulting indexes are broadly applicable for downstream analysis, so it makes sense to share them. However, there is no simple tool to do this. To this end, we introduce refgenie, a reference genome assembly asset manager. Refgenie makes it easier to organize, retrieve, and share genome analysis resources. In addition to genome indexes, refgenie can manage any files related to reference genomes, including sequences and annotation files. Refgenie includes a command-line interface and a server application that provides a RESTful API, so it is useful for both tool development and analysis.

Availability: `https://refgenie.databio.org`

Background

Enormous effort goes into assembling and curating reference genomes[1–5]. These reference assemblies provide a common representation for comparing results and they form the basis for a wide range of downstream tools for sequence alignment and annotation. Many tools that rely on reference assemblies will produce independent resources that accompany an assembly. For instance, many aligners must *hash* the genome, creating *indexes* that are used to improve alignment performance[6–9].

Analytical pipelines typically rely on these aligners and their indexes for the initial steps of a data analysis. These assembly resources are typically shared among many pipelines, so it's common for a research group to organize them in a central folder to prevent duplication. In addition to saving disk space, centralization simplifies sharing software that uses a reference assembly because software can be written around a standard folder structure. However, this does not solve the problem of sharing genomic resources *between* research groups. Because each group may use a different strategy to identify shared genome resources, sharing tools across groups may require modifying them.

One solution to this problem is to have a web-accessible server where standard, organized reference assemblies are available for download. Indeed, this is exactly the goal of Illumina's *iGenomes* project, which provides "a collection of reference sequences and annotation files for commonly analyzed organisms"[10]. The iGenomes project has become a popular source of genome assets and has greatly simplified sharing analysis tools among research environments. However, this approach suffers from some fundamental drawbacks and leaves several challenges unsolved. First, the individual assets can only be downloaded in bulk, but what if a particular use case requires only a small subset of resources in a package? More important, building the resources is not scripted, so if the repository excludes a reference or resource of interest, there is no programmatic way to fill the gap. In these scenarios, users must manually build and organize genome assets individually, forfeiting the strength of standardization among groups.

To improve the ability to share interoperable reference genome assets, we have developed *refgenie*, which enables a more modular, customizable, and user-controlled approach to managing reference assembly resources. Like iGenomes, refgenie standardizes reference genome asset organization so software can be built around that organization. But unlike iGenomes, refgenie also automates the *building* of genome assets, so that an identical representation can be produced for any genome assembly. Furthermore, refgenie allows programmatic access to individual resources both remote and local, making it suitable for the next generation of self-contained pipelines.

Refgenie can organize any files that can be assigned to a particular reference genome assembly, which could include not only genome indexes, but other resource types

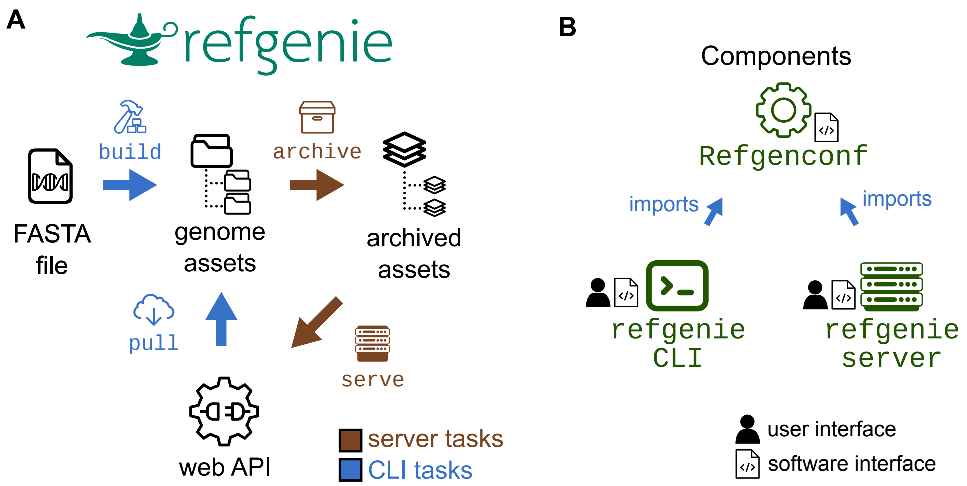

*Fig. 1: **Refgenie concept and software organization**. A: Refgenie provides the ability to either build or pull assets. B: Refgenie is tripartite, made up of a `conf` utility, a command-line interface (CLI), and a server package. The configuration package is intended for programmatic use, and is used by the CLI and server packages. Users and software use refgenie via the CLI or server (web API).*

like genome sequences and annotations[11–13].

Refgenie manages genome-related resources flexibly. It can handle any asset type, from annotations to indexes. It provides individual, pre-built asset downloads from a server and allows scripted building for custom inputs. Refgenie thus solves a major hurdle in biological data analysis.

Results and discussion

Refgenie is the first full-service *reference genome asset manager*. Refgenie provides two ways to obtain genome assets: *pull*, and *build* (Fig.1A). For common assets, *pulling* a pre-built version obviates the need to install and run specialized software to build a particular asset. It also makes it easier to satisfy prerequisites programmatically for pipelines and other software. However, remote-hosted assets are only practical for common genomes and assets, so for uncommon assets or on unconnected computers, users may instead *build* assets, which creates the same standard output for custom genomes. By providing both *build* and *pull*, refgenie facilitates asset organization both within and between research groups, increasing interoperability of tools that rely on genome resources.

Asset organization

Refgenie uses a local `YAML` file called the *genome configuration file* (Fig. 4) to keep track of metadata, such as local file paths. In this file, refgenie stores paths to individual genome assembly resources, or *assets*, each of which represents one or more files. You can think of a genome asset as a folder of related files tied to a particular genome assembly. For example, an asset could be an index for a particular tool, or a group of annotation files. Refgenie assets are referred to using *asset registry paths*, which are human-readable asset identifiers. The registry path follows the structure `{genome}/{asset}:{tag}`; a genome thus operates as a sort of namespace for a set of assets, which are identified both by asset names as well as tags, allowing refgenie to manage multiple versions of the same asset.

The `refgenie` software suite allows users to interact with assets with three components: 1) a command-line interface (CLI), 2) a server, and 3) a configuration package that supports them both (Fig.1B).

Refgenie command-line interface

The workhorse of refgenie is the command-line interface (CLI); it is how users will typically interact with genome assets. Its implementation as a command-line tool not only makes it useful for general purpose exploration and access, but also allows it to be integrated into existing workflows that require access to genome assets from the shell. The CLI can be installed with `pip install refgenie` and invoked by calling `refgenie`. The refgenie CLI provides 7 functions for interacting with local genome assets:

- `refgenie init` – initializes an empty genome configuration file
- `refgenie list` – summarizes the genome configuration file, listing local genomes and assets
- `refgenie seek` – provides the file path to a given asset
- `refgenie add` – adds an already-built local asset
- `refgenie remove` – removes a local asset
- `refgenie tag` – adds a tag to a local asset
- `refgenie build` – builds a new asset

asset name	genome	asset size	archive size	build time	peak memory
fasta	hg38	2.9 GB	0.8 GB	0:01:17	0 GB
bowtie2_index	hg38	3.9 GB	3.5 GB	0:57:29	5.6 GB
hisat2_index	hg38	4.2 GB	3.9 GB	0:36:22	5.5 GB
bismark_bt1_index	hg38	13.6 GB	7.5 GB	1:10:24	10.8 GB
bismark_bt2_index	hg38	13.6 GB	7.5GB	2:17:22	10.8 GB
bwa_index	hg38	2.9 GB	3.2 GB	0:51:02	4.7 GB
star_index	hg38	26.9 GB	24.3GB	1:51:11	35.8 GB
kallisto_index	hg38_cdna	2.2 GB	1.6 GB	0:04:30	3.8 GB
salmon_index	hg38_cdna	3.1 GB	2.6 GB	0:03:04	5.3 GB
dbnsfp	hg38	22.9 GB	22.8 GB	2:35:30	0 GB*
ensembl_rb	hg38	0.01 GB	0.01 GB	0:00:01	0 GB
ensembl_gtf	hg38	0.06 GB	0.06 GB	0:00:13	0 GB
gencode_gtf	hg38	0.04 GB	0.04 GB	0:00:01	0 GB
feat_annotation	hg38	0.01 GB	0.01 GB	0:01:55	0.1 GB
refgene_anno	hg38	0.03 GB	0.01 GB	0:00:30	0.2 GB

*Fig.2: **Assets available for build**. Table listing assets that can currently be built with `refgenie build`, along with statistics for size, build time, and memory high water mark. Assets were built for the human genome using a single core. Times and memory are representative values from a single run. These assets are produced by various tools[8,9,14–17] and are available to be built for any arbitrary genome input. * peak disk space usage for dbnsfp is over 300GB*

Initializing refgenie

All of the CLI commands require knowledge of the refgenie configuration file, which is passed via the `-c` argument. To install and configure refgenie requires only a few lines of code:

```
pip install --user refgenie
export REFGENIE="refgenie.yaml"
refgenie init -c $REFGENIE
```

In this example, we populate the `$REFGENIE` environment variable, which eliminates the need to pass `-c` to each command going forward. The `init`, `list`, `add`, and `remove` functions are relatively straightforward and simply allow a user to create, view, and manipulate the genome configuration file.

Building assets

The `build` function allows a user to *build* assets for any arbitrary inputs, which is what enables refgenie to serve custom genomes. Refgenie has built-in capability to build a selection of different common genome assets (Fig.2). The list of assets with available recipes are listed by the `refgenie list` command. Available assets are built by specifying the asset registry path along with any required inputs. For example:

```
refgenie build hg38/ASSET \
  --INPUT FILE
```

Where `ASSET` is a unique key defining the asset of interest (*e.g.*, `bowtie2_index`), `INPUT` is an identifier for a required input, and `FILE` is a path to the provided input. For example, to build a `fasta` asset requires a compressed fasta file as input. It can thus be built like this:

```
refgenie build hg38/fasta \
  --fasta hg38.fa.gz
```

Building an asset can require either input arguments, such as in this example, or it can require other assets. The list of requirements for building an asset can be found by adding the `-r` argument to the `build` function. Assets are built with locally available versions of the software (*e.g.* `bowtie2-build` to create the Bowtie2 index) or alternatively with containerized software (using `-d/--docker` flag in the `refgenie build` command). We have also produced a complete containerized computing environment capable of building all available refgenie assets, which can be deployed with the *bulker* environment manager[18], making it easy to build any refgenie assets without installing the required tools natively.

Pulling assets

In addition to functions on local assets, the refgenie CLI also contains additional commands that can interact with remote assets: *pull* and *listr*:

- `refgenie listr` – lists available remote genomes and assets
- `refgenie pull` – downloads a remote asset

With these commands, refgenie downloads a standard asset with a single line of code:

```
refgenie pull hg38/ASSET
```

Tagging assets

The `tag` command allows users to tag assets with unique identifiers. Tags may also be provided when building or pulling assets to specify a version (*e.g.* `build hg38/ASSET:TAG`). Once tagged, specific versions of

assets can be accessed by tag. If no tag is specified, refgenie will use the tag `default`, which is automatically given to any built or pulled assets that do not specify a tag. This makes tags an optional feature of refgenie which are only necessary if a user desires multiple versions of the same asset.

Seeking assets

Once the asset has been added to refgenie either via pulling or building, the user can retrieve the path to it with `refgenie seek`:

```
refgenie seek hg38/ASSET
```

This command returns the file path to the specified asset for the specified genome. The `seek` command is portable, eliminating the need to hard-code paths or pass them as arguments. Consequently, in a pipeline or other software that requires access to genome assembly assets, the path to the local `bowtie2_index` asset can be retrieved with a shell command:

```
bowtie2_index_path=\
  $(refgenie seek hg38/bowtie2_index)
```

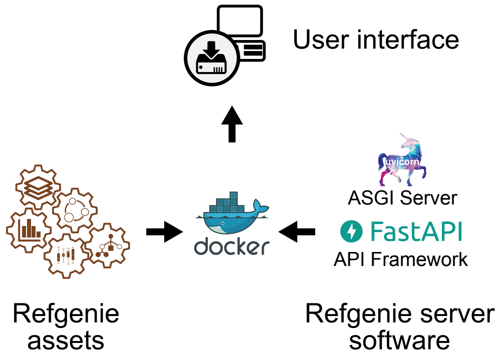

*Fig.3: **Server software stack.** Archived refgenie assets are mounted into a Docker container, along with the refgenie server software, which is built using FastAPI and uvicorn. The container can then be accessed via the web and API user interfaces.*

```
genome_folder: /genomes/path
genome_server: http://...
config_version: 0.3
genomes:
  hg38:
    assets:
      bowtie2_index:
        asset_description: ...
        default_tag: default
        tags:
          default:
            asset_path: bowtie2_index
            asset_digest: 0f9217d44264ae2188
            seek_keys:
              bowtie2_index: .
```

*Fig.4: **Genome config file.** Refgenie reads and writes a genome configuration file in YAML format to keep track of available local assets.*

Refgenie server

The `listr` and `pull` functions require that the CLI interact with a server. The CLI uses a configurable URL to retrieve a remote archived tarball. After retrieving the tarball, the CLI will unpack it into the appropriate folder location and update the configuration file to provide access to its path via `refgenie seek`.

To support this remote function, we have developed a containerized, portable, open-source companion application called `refgenieserver`. Many users of refgenie will not have to be aware of the server application; however, interested users can use refgenie server to host their own genome asset server. For example, a tool developer may wish to simplify use by hosting indexes for common reference assemblies.

Running the refgenie server is simple for users who are already familiar with refgenie. It reads the same genome configuration file format as the CLI. In fact, refgenie server operates on the same genome config file and asset folders that that refgenie itself builds or downloads. The server software comes with an `archive` command that prepares a refgenie genome folder for serving. It compresses each asset into an individual tarball. This simple system makes it easy for users to run a server using their refgenie assets.

This server software leverages cutting-edge web technology to provide high-concurrency service with minimal compute resources (Fig. 3). We built refgenie server on top of the FastAPI Python framework, which is a high performance web framework for building APIs. FastAPI automatically produces an API that complies with OpenAPI 3.0 standards, which will allow other tools to discover and automatically use the API. It also includes a self-documenting test interface so that users can see and test the available API endpoints. Refgenie leverages the Starlette development toolkit and the `uvicorn` server to make use of the high-performance Asynchronous Server Gateway Interface (ASGI) specification, which provides asynchronous access to refgenie server.

Refgenie server is containerized and available on dockerhub, so that an interested user could run a server with a single line of code:

```
docker run --rm -p 80:80 \
  -v genomes_folder:/genomes rgimage \
   refgenie -c /genomes/config.yaml serve
```

By mounting a refgenie 'genomes' folder into this container, users get a fully functioning web interface and RESTful API.

Refgenconf package for genome configuration

Refgenie organizes assets by genome in the configuration file, which is both computer-readable and human-readable. In practice, users will not need to interact with this file at all, as refgenie will handle both reading and writing the file. However, users may edit the file if they need a more complicated structure (such as storing assets on different file systems, or adding assets manually). Together with the refgenie software, this simple file makes the concept of reference genome assets completely portable. Full documentation for the configuration file format can be found at refgenie.databio.org.

The configuration package, `refgenconf`, simply provides functions and data types to read and write items listed in the genome configuration file. Under the hood, the refgenie CLI itself uses `refgenconf` to interact with the genome configuration and assets on disk. The server software also relies on it to read, archive, and serve assets. The `refgenconf` package also provides the starting point for any third-party Python developers by providing a fully functional Python application programming interface (API) for interacting with refgenie assets. For example, we use `refgenconf` in Python pipelines we develop to make them aware of the genome assets available in a given computing environment. Using this approach, a pipeline need only be provided with an assembly key, like 'hg38', and it can use `refgenconf` to locate the correct path to any genome-related asset necessary for the pipeline. This simplifies the process of configuring pipelines and allows `refgenie` to be used both by humans and computers.

The Refgenomes database

We designed the server software so that anyone could easily run a custom server instance. We have also deployed our own instance of `refgenieserver` at refgenomes.databio.org, where we host pre-built genome assets. Like any instance of `refgenieserver`, our refgenomes database provides both a web interface and a RESTful API to access individual assets we have made available. Users may explore and download archived indexes from the web interface or develop tools that programmatically query the API.

The web interface provides a graphical listing of available genomes and assets, allowing users to browse the site and download individual assets manually. In addition, refgenieserver provides API endpoints to serve lists of available genomes and assets, as well as metadata for the individual assets, including unique digests for file integrity, file sizes, and archive content information. Furthermore, the server provides endpoints to download each asset individually. Endpoints include the following: `/genomes` retrieves a list of available genomes; `/assets` retrieves a list of all available assets; `/{genome}/assets/` retrieves a list of assets for a given genome; and `/{genome}/assets/{asset}/archive` retrieves the tarball for the specified asset. Complete documentation is available at refgenomes.databio.org. Because it provides a standard OpenAPI-compliant RESTful API, our server will be useful not just for our refgenie CLI, but for other tools that would benefit from automated access to reference assembly assets and indexes.

Our `refgenieserver` instance runs within DC/OS as a containerized application. The server application makes genome assets available through a web application connected directly to a remote filesystem, with no additional database or infrastructure requirements. Integration and deployment is automated using GitHub, Travis-CI, Docker Hub, and a custom deployment technique made simple in DC/OS. Changes committed in code are generally deployed to development or production services within 1-3 minutes.

Genome provenance

One challenge with genome assembly assets is name mismatches that lead to analysis conflicts. Because refgenie identifiers are human-readable and user-controlled, refgenie cannot rely on them to uniquely identify assets. Furthermore, refgenie assets may be either built or pulled from different servers, exacerbating the issue. This is an active area of research, with several approaches under development related to this problem, such as the NCBI Assembly database[4], the refget protocol for sequence identifiers[19], and tximeta checksums for RNA-seq data[20]. Refgenie currently provides two resources to confirm the identity of pulled and built assets: First, a unique digest for each asset, and second, a building log file. Refgenie makes unique asset digests available via both web interface and API, allowing users to distinguish between two assets with the same names but different digests. Furthermore, because building refgenie assets is scripted, it is possible to trace any asset back to its inputs. Refgenie server provides API points to retrieve either the raw recipe (`/v2/asset/{genome}/{asset}/recipe`) or the actual log file (`/v2/asset/{genome}/{asset}/log`) for any asset available on the server. For built assets, the `build` command automatically produces a log file that records the input files, software versions, and final digests for any locally built assets. These resources make it possible for users to uniquely identify and trace the provenance of assets they either build or pull.

Comparison to existing tools

Refgenie fills a niche for which, to our knowledge, there is no other competing software. The most similar projects are Illumina's *iGenomes* and Galaxy Data Managers accompanied by Galaxy Tool Shed[21,22], both of which offer only a small part of what refgenie does

	web interface to download assets	modular access to individual assets	custom genomes	RESTful API for assets	command-line interface and asset manager	containerized server software	python API
Refgenie	✓	✓	✓	✓	✓	✓	✓
iGenomes	✓	✗	✗	✗	✗	✗	✗
Data managers	✓	✓*	✓	✗	✗	✗	✗

*Fig.5: **Feature comparison**. iGenomes and Galaxy Data managers also solve the problem of standardized reference genome assets, but both lack the interactive features of refgenie. *Data managers assets can be accessed individually, but not outside of the Galaxy UI.*

(Fig. 5). iGenomes provides a single archive download of a standardized folder structure with pre-build assets for pre-defined genomes. The Data Managers facilitate building of assets but require a Galaxy server as well as administrator access. Furthermore, there is no generic way to retrieve the paths to the assets outside of the Galaxy server UI. Some of refgenie's utility is also satisfied by individual tool websites that provide asset downloads (*e.g.* bowtie2 indexes), but these provide no shared structure or unified interface for access.

In contrast, refgenie provides a full-service manager that unifies and transcends all of these available tools. Refgenie solves a series of related problems all in one convenient package. It provides a unified web interface for all assets, plus programmatic access to modular individual assets via a RESTful API for metadata and assets. Refgenie also provides the ability to build assets for custom genomes with a uniform interface that integrates seamlessly with downloaded assets. Refgenie is unique in providing a local asset manager that makes locating assets portable, simplifying building pipelines that use these assets. It is also the only easily deployable, independent, containerized server application and Python API for reference genome assets. Thus, no existing software can solve these problems specific to genome-related data resources.

Conclusions and future directions

Reference genomes, indexes, annotations, and other genome assets are integral to sequencing analysis projects, and these genome-associated data resources are growing rapidly[11]. Refgenie provides a full-service management system that includes a convenient method for downloading, building, sharing, and using these resources. Refgenieserver is among a growing number of API-oriented projects in the life sciences[5,23,24]. Refgenie will simplify management of reference assembly assets for users and groups, facilitating data sharing and software interoperability[25].

Several new features under development will make refgenie even more useful. Currently, refgenie is completely flexible with respect to genomes, but it is less flexible with respect to assets, as only pre-scripted assets can be built. A more flexible approach would allow refgenie to accept custom recipes, allowing users to add new asset types. Future development will address the challenges of sharing recipes, provenance, and trust for flexible assets. We are also improving the way refgenie records and uses identifiers and relationships among assets. For instance, by recording more detailed information about what an asset contains and how it was generated, we open the possibility of delineating more fine-grained compatibilies. For instance, while two indexes would only be compatible if derived from the same set of sequences, two annotation files could be compatible on different sequences that shared a coordinate structure. Finally, we anticipate that future development will extend refgenie to be able to accommodate ontology annotation for assets and genomes. Together, these improvements will enable more robust discovery of assets and genomes as well as the relationships among them.

Software availability

Refgenie, refgenieserver, and refgenconf Python packages are all BSD2-licensed. Source code and documentation can be found at refgenie.databio.org. Refgenie is registered at SciCrunch (SCR_017574) and bio.tools (biotoolsID:Refgenie).

References

1. Harrow, J. *et al.* GENCODE: The reference human genome annotation for the ENCODE project. *Genome Research* **22,** 1760–1774 (2012).

2. Pruitt, K. D., Tatusova, T., Brown, G. R. & Maglott, D. R. NCBI reference sequences (RefSeq): Current status, new features and genome annotation policy. *Nucleic Acids Research* **40,** D130–D135 (2011).

3. Church, D. M. *et al.* Modernizing reference genome assemblies. *PLoS Biology* **9,** e1001091 (2011).

4. Kitts, P. A. *et al.* Assembly: A resource for assembled genomes at NCBI. *Nucleic Acids Research* **44,** D73–D80 (2015).

5. Ruffier, M. *et al.* Ensembl core software resources: Storage and programmatic access for DNA sequence and genome annotation. *Database* **2017,** (2017).

6. Sadakane, K. & Shibuya, T. Indexing huge genome sequences for solving various problems. *Genome Informatics* **12,** 175–183 (2001).

7. Hon, W.-K., Sadakane, K. & Sung, W.-K. Breaking a time-and-space barrier in constructing full-text indices. *SIAM Journal on Computing* **38,** 2162–2178 (2009).

8. Li, H. & Durbin, R. Fast and accurate short read alignment with burrows-wheeler transform. *Bioinformatics* **25,** 1754–60 (2009).

9. Langmead, B. & Salzberg, S. L. Fast gapped-read alignment with bowtie 2. *Nat. Methods* **9,** 357–359 (2012).

10. Illumina. IGenomes. Ready-to-use reference sequences and annotations. *support.illumina.com* (2019).

11. Richa Agarwala *et al.* Database resources of the national center for biotechnology information. *Nucleic Acids Research* **46,** D8–D13 (2018).

12. Zerbino, D. R., Wilder, S. P., Johnson, N., Juettemann, T. & Flicek, P. R. The Ensembl Regulatory Build. *Genome Biology* **16,** (2015).

13. Sheffield, N. C. & Bock, C. LOLA: Enrichment analysis for genomic region sets and regulatory elements in R and bioconductor. *Bioinformatics* **32,** 587–589 (2016).

14. Krueger, F. & Andrews, S. R. Bismark: A flexible aligner and methylation caller for bisulfite-seq applications. *Bioinformatics* **27,** 1571–1572 (2011).

15. Bray, N. L., Pimentel, H., Melsted, P. & Pachter, L. Near-optimal probabilistic RNA-seq quantification. *Nature Biotechnology* **34,** 525–527 (2016).

16. Kim, D., Langmead, B. & Salzberg, S. L. HISAT: A fast spliced aligner with low memory requirements. *Nature Methods* **12,** 357–360 (2015).

17. Dobin, A. *et al.* STAR: Ultrafast universal RNA-seq aligner. *Bioinformatics* **29,** 15–21 (2012).

18. Sheffield, N. C. Bulker: A multi-container environment manager. *OSF Preprints* (2019). doi:10.31219/osf.io/natsj

19. GA4GH. Refget - reference sequence retrieval implementation. *samtools.github.io/* (2019).

20. Love, M. I. *et al.* Tximeta: Reference sequence checksums for provenance identification in RNA-seq. *bioRxiv* (2019). doi:10.1101/777888

21. Blankenberg, D., Johnson, J. E., Taylor, J. & Nekrutenko, A. Wrangling galaxy's reference data. *Bioinformatics* **30,** 1917–1919 (2014).

22. Blankenberg, D. *et al.* Dissemination of scientific software with galaxy ToolShed. *Genome Biology* **15,** 403 (2014).

23. Yates, A. *et al.* The ensembl REST API: Ensembl data for any language. *Bioinformatics* **31,** 143–145 (2014).

24. Tarkowska, A. *et al.* Eleven quick tips to build a usable REST API for life sciences. *PLOS Computational Biology* **14,** e1006542 (2018).

25. Wilkinson, M. D. *et al.* The FAIR guiding principles for scientific data management and stewardship. *Sci. Data* **3,** 160018 (2016).

Personal Cover

Click here to access/download;Personal Cover;response_to_reviewers.pdf

Dear Editor

Thank you for the positive report. We have now completed a thorough response to all the reviewer concerns. We just released a major new update of the refgenie software which includes a lot of new features, including some prompted by the reviewer comments as well as other features we have been working on. Our paper is reorganized and re-written to address some confusion raised by the reviewer comments. Notably, as requested, we added some discussion on provenance and trust and also added some new features to help users identify and track their assets. We also added a future directions section and a comparison to galaxy tools. Refgenie is now registered at SciCrunch (SCR_017574) and bio.tools (biotoolsID:Refgenie), as now mentioned in the software availability section. This revision has improved refgenie and the paper and we are excited to re-submit it for your consideration.

Sincerely,

Nathan Sheffield

Nathan Sheffield, PhD, on behalf of all co-authors
Assistant Professor, Center for Public Health Genomics, University of Virginia
www.databio.org
434-924-8278

Dear Dr. Sheffield,

Your manuscript “Refgenie: a reference genome resource manager” (GIGA-D-19-00289) has been assessed by our reviewers. Based on these reports, and my own assessment as Editor, I am pleased to inform you that it is potentially acceptable for publication in GigaScience, once you have carried out some minor revisions suggested by our reviewers.

Their reports are below. I feel the reviewers (Andrew Yates, Katherine James, Bernie Pope and Christophe Dessimoz) make useful suggestions that will improve the manuscript, including for example the suggestion to discuss future directions in more detail. I also think that reviewer 3 raises an interesting point regarding “provenance and trust” of genome assets that are shared via servers. Also, please compare your tool to other (e.g. Galaxy) applications that are somewhat similar, as mentioned in the reviews.

In addition, please register any new software application in the bio.tools and SciCrunch.org databases to receive RRID (Research Resource Identification Initiative ID) and biotoolsID identifiers, and include these in your manuscript. This will facilitate tracking, reproducibility and re-use of your tool.

Please also make sure your revised manuscript is formatted according to our guidelines for “Technical Notes”, including a “software availability” section.

Reviewer #1:

Recommendation: Accept with possible minor modifications

Review

The manuscript describes a single tool for the management and distribution of genome related assets for use in downstream analysis, such as variant calling and RNA-seq quantification. Refgenie is both a python binary for assets management on a local system and a web API for centralising and disseminating said assets, which can be used independently of the refgenie binary. The tool is both novel and useful capable of an immediate impact within any analysis workflow. As such I believe the manuscript should be accepted and raise only a set of minor points detailed below.

Thank you!

1) Comparison of refgenie to iGenomes

As stated in the manuscript, the iGenomes resource is one of the few resources which attempts to provide similar functionality. One aspect missed out of the comparison between the two is that iGenomes covers a much wider number of genomes and annotation sets than the reference deployment at refgenomes.databio.org.

Documentation at refgenie.databio.org has information on how a researcher could host iGenomes data within refgenie but would the authors be open to mirroring the entire contents of iGenomes on their reference implementation or to create a standard import module to help users import an entire iGenomes file in a single command. I feel that would help drive adoption and remove the last point here iGenomes still has an upper hand over refgenie.

> *To address this, we have created a new command line tool called `import_igenome` that is installed along with `refgenie`. It adds all the assets enclosed in the genome archive downloaded from the iGenomes website to the Refgenie local asset inventory. The required inputs are: * `-g`: name of the genome that should be assigned to the assets, * `-p`: a path to the downloaded archive or a directory (unarchived archive).*
>
> *We have added detailed documentation as well.*
>
> *usage:*

```
$ import_igenome -h

usage: import_igenome [-h] -p PATH -g GENOME [-c CONFIG]

Integrates every asset from the downloaded iGenomes tarball/directory with
Refgenie asset management system

optional arguments:
  -h, --help            show this help message and exit
  -p PATH, --path PATH  path to the desired genome tarball or directory to
                        integrate
  -g GENOME, --genome GENOME
                        name to be assigned to the selected genome
  -c CONFIG, --config CONFIG
                        path to local genome configuration file. Optional if
                        'REFGENIE' environment variable is set.
```

> *Example:*

```
$ import_igenome -g staph -p Staphylococcus_aureus_NCTC_8325_NCBI_2006-02-13.tar.gz
Moved 'Staphylococcus_aureus_NCTC_8325_NCBI_2006-02-13.tar.gz' to '/Users/mstolarczyk/Desktop/test
Added assets:
- staph/Chromosomes
- staph/BWAIndex
- staph/BowtieIndex
- staph/AbundantSequences
- staph/Bowtie2Index
- staph/WholeGenomeFasta
```

2) Lack of future directions and detailing of the limitations of the current refgenie implementation

The manuscript does not detail the areas and enhancements refgenie would look towards implementing in the future. This for me would include a). how to easily inject new asset creation methods into the build process e.g. if a new tool appeared that gained popularity how would that be supported b). steps refgenie will take to ensure portability of genome identifiers across resources and ensuring that these resources are the same.

> *Thank you for the suggestion. We have added 2 new sections to the manuscript. First, we now have a "Genome provenance" section, where we describe how the current implementation handles this. In fact, we have already made substantial progress here and the current version goes partway toward solving this problem. In addition to this, we have also added a section called "Future directions", as requested. In this section we discuss a few problems that we are actively working on and expect to identify new solutions in the future. Specifically, we outline 3 areas for future development: First, adding new assets, as you mention; second, dealing more robustly with the genome provenance issues; and finally, adding in ontology metadata to individual genomes and assets.*

On this second point I should disclose that the corresponding author, Nathan Sheffield, has recently joined a Global Alliance for Genomics and Health project called refget, which I head up. I know that the work he is participating on will help to resolve the issue of genome identity across resources. Whilst I am not looking for a namecheck here, I do feel the authors should flag this as a potential issue and that steps are being taken to address it.

Overall though the manuscript is excellent and I congratulate the authors on their work.

Andrew Yates EMBL-EBI

> *Thanks, we are looking forward to continuing to explore how refgenie and refget can build on one other to solve these problems! We have already started by integrating refget checksums into the refgenie system, which can be useful to some users. We think that this idea is ready for lots of future expansion and we aren't yet making total use of the power this will eventually enable.*

Reviewer #2:

Review

Refgenie is a very useful tool that has several functions that are not provided by iGenomes. In particular, it allows the use of custom genomes, which will become increasingly necessary as researchers work with more diverse species following recent large genome sequencing projects, such as the International Vertebrate Sequencing Project. Refgenie would also be of particular use to groups working with multiple bacterial strains, where a large number of reference genomes may be in use.

The manuscript is extremely well written and describes the architecture and usage of Refgenie. I have four minor comments:

1)

The results section introduces the three components of Refgenie: 1) command-line interface, 2) server, 3) configuration package but discusses them in the following subsections in a different order, and after the "Genome configuration and asset organisation" section. From the readers point of view, reordering these sections to describe these three components in this order would aid clarity. Indeed, the "command-line interface" section is likely to be of most interest to potential users of Refgenie. Additionally, as a suggestion only, combining the "Genome configuration and asset organisation" with "Refgenconf configuration package" would also aid clarity.

> *Suggested amendments have been made to the text. Thank you for the suggestion!*

2)

The "command line interface" section provides some example commands for a download of a bowtie2 index for hg38. For better understanding of refgenie usage, it would be useful if it also had similar real world examples for the other local genome assets commands, for instance a build for a local resource.

> *An example of `refgenie build` command has been added to the text. In fact, we've greatly expanded this small tutorial to try to make it easier to understand. There's also now much more thorough documentation on the website.*

3)

In the same section, a real example of how the refgenie seek command is portable and eliminates the need for hard coded paths would aid understanding of this concept.

> *An illustration of the `refgenie seek` command portability has been added to the text, and also to the web documentation.*

4)

In the section, "comparison with existing tools" the authors state that iGenomes is only available as a single archive download. However, it should be noted that there are other sources for download of indexed reference genomes. For instance pre-built indexes for model organisms are available individually from the bowtie2 website and via the command line from the linked ftp site. Additionally, the Galaxy Tool Shed provides data managers for index builds for multiple tools. This section should be expanded to highlight the unique features provided by Refgenie over existing resources for genome assets.

> *The "Comparison to existing tools" section has been expanded and now references Galaxy Tool Shed. Furthermore, we now use this section to highlight unique features of refgenie, as suggested.*

Reviewer #3:

Review

This paper describes Refgenie a system for managing and distributing genome reference files and their associated assets (such as index files etc).

Managing genome references and their associated files is a common problem faced by bioinformaticians and Refgenie provides a potentially useful service in this area. Refgenie is based on a server system for storing and sharing reference data, and a command line interface for interacting with the server. A user can request a copy of an existing reference resource from the server, or if that does not already exist, they can supply a new FASTA file and ask Regenie to automatically create a new reference resource from that file.

Sharing and managing genome reference data can be useful within research groups and organisations, where consistency in analyses is important.

Overall this is a well written and tidy paper that describes a potentially useful tool that may be of interest to the journal readership.

I have a few comments about the paper that I believe should be addressed before publication:

1)

An important issue with sharing and reusing genome assets is provenance and trust. I can foresee that users will want some level of certification about who has supplied the genome and how it was processed. For instance, perhaps genome assets could use public key cryptography to sign the data and provide a level of certainty about its origin? In short, how can we trust the data that we get back from Refgenie?

We have addressed this concern with a new section called "genome provenance". Here, we discuss the way refgenie guarantees asset identity. Furthermore, we have also added a new capability to the server, whereby users can see the exact log outputs generated for each asset. This is one of the benefits of refgenie: assets are scripted, so we can trace exactly what they are. Refgenie also records a checksum of the final built asset. The refgenie CLI uses this checksum to confirm that the identity of a pulled asset matches the remote asset. We have added a new section to the manuscript and documentation that describes this feature. We are also working with the refget team to come up with universal ways to address this problem more generally, in terms of not just assets but genome assemblies themselves – we have mentioned this now in the future directions section.

2)

The authors suggest that there is little prior work for comparison, which is probably true. However, I think one possible partial competitor is the CVMFS system used by the Galaxy project, which has been used to share reference data resources, e.g. https://training.galaxyproject.org/training-material/topics/admin/tutorials/cvmfs/tutorial.html. I think it is worth comparing Refgenie to CVMFS.

Thanks for the suggestion, we now reference the Galaxy Data Managers/Tool Shed in the "Comparison to existing tools" section. To describe the relationship, although Galaxy-CVMFS aims to address the same issue as Refgenie, its scope is more similar to the iGenomes project by Illumina. CVMFS is a distributed filesystem for sharing read only data that is used by Galaxy software to provide a wide array of reference genomes and assets required for analyses with the tool. A potential user can mount and configure CVMFS locally using Ansible (introduces additional software requirement) or by hand (a multi-step process). The resources are located in two parallel and self-contained directories: /indexes and /managed. The former, which is manually curated, stores the assets in a similar manner to Refgenie (genome/asset), but the structure is not standard (see discrepancies in: http://datacache.galaxyproject.org/indexes/hg38/ and http://datacache.galaxyproject.org/indexes/hg19/), which makes seamless reference genome switching impossible in the pipelines that were built around this directory structure. Moreover, there is no way to add assets for custom reference assemblies. The /managed directory is created by Data Managers that use a combination of Tool Data Tables and .loc files to keep track of the available reference assemblies and assets. A newly downloaded/built asset can be subsequently used for the analyses on the Galaxy server and potentially outside, in a custom pipeline. The notion of Data Managers adds flexibility to the genome assets management but not only requires the Galaxy server installed but also a Galaxy Administrator access. Furthermore, there is no generic way to retrieve the paths to the assets outside of the Galaxy server UI.

In any case, it's clear that there's some similarity and we've added a discussion around this, but refgenie is filling a niche that is not filled by the CVMFS system.

3)

On page 3 I found it slightly difficult to understand how the build command works. Where does it run? How does it know what to run? Can the user control the versions of tools which are run?

The build command is more complex than the pull command because it requires additional inputs. We have added detail and examples to make this more clear. At the moment, refgenie is flexible with respect to genomes, but not with respect to assets to build, which are hard-coded into refgenie. The build process runs on the user's computer locally, so it uses whatever versions are available in the user's PATH. Refgenie uses a simple recipe system that describes how assets are built. While an experienced user could relatively easily add a new asset recipe (it's simply a python dict in the code), or customize versions of tools, this is a clear area for

future development. We now present this in the discussion as a future direction to build a custom recipe system. Furthermore, we have added more detailed information to the web documentation.

4)

I'm not sure that "lightning fast" is an appropriate adjective for a scientific paper.

The expression has been corrected. This is how uvicorn self-describes their software, which is how it got in there in the first place, but you're right.

5)

On page 5 there is quite a lot of technical detail about the implementation of the refgenie server. I'm not sure that this level of detail is required for the paper.

We have reduced the detail here. Our goal was just to demonstrate the robustness of the server and describe an example for hardware others could use to deploy their own refgenieserver instance.

6)

Please use "Python" (proper noun) for the programming language instead of "python".

The typos have been corrected.

7)

I tried to use Refgenie on the HPC system where I work. However, I ran into a problem:

```
$ python3 --version
Python 3.7.1

$ python3 -m venv refgenie_dev
$ source refgenie_dev/bin/activate
(refgenie_dev)$ refgenie
Traceback (most recent call last):
  File "/home/foo/scratch/refgenie_dev/bin/refgenie", line 11, in <module>
    load_entry_point('refgenie==0.6.0', 'console_scripts', 'refgenie')()
  File "/home/foo/scratch/refgenie_dev/lib/python3.7/site-packages/refgenie/refgenie.py", line 387
    parser = logmuse.add_logging_options(build_argparser())
  File "/home/foo/scratch/refgenie_dev/lib/python3.7/site-packages/refgenie/refgenie.py", line 98,
    sps[BUILD_CMD], groups=None, args=["recover", "config", "new-start"])
  File "/home/foo/scratch/refgenie_dev/lib/python3.7/site-packages/pypiper/utils.py", line 60, in
    argument_groups=groups, arguments=args, use_all_args=all_args)
  File "/home/foo/scratch/refgenie_dev/lib/python3.7/site-packages/pypiper/utils.py", line 808, in
    from logmuse import LOGGING_CLI_OPTDATA
```

I tried other versions of Python 3, but still had the same problem.

This was a temporary incompatibility introduced in one of refgenie dependencies, `pypiper`. It was resolved after being reported on Aug 28th: https://github.com/databio/refgenie/issues/110 – sorry for the inconvenience.

We have also now released version 0.7.0 of refgenie. Please give this another try!

Reviewer #4:

Review

The submission present a system called "refgenie" to build, store, and share genomes and associated indices, which are often required by downstream processes. The authors argue that this is a unique niche, the closest related system being Illumina's iGenomes.

I agree that the practical problem tackled is of high interest, and that the software proposed is a promising solution to that problem. The manuscript is clearly written and nicely showcases the high-level features. This is complemented by extensive documentation and tutorials, neat code, and a highly user friendly installation procedure through PyPI.

In particular, the flexibility of supporting remote retrieval of data via CLI, API and a web interface makes refgenie a compelling solution.

I have no major or minor reservation, just a few discretionary points to the authors.

1)

One alternative would be to use git lfs for the storage of genomic data. This could be added to the comparison table (with appropriate amendements to the columns, if sensible)

> *It is true that git lfs can be used to distribute data that can be accessed via CLI; in fact there are many technologies that could be used, such as an FTP server, Amazon S3, etc. These systems are more like underlying technology upon which an API system like refgenie could be built, rather than something that can compare against refgenie. In other words, we could have built refgenie on something like git lfs, for the actual transfer of files. As such, we don't think the comparison to generic file distribution systems would be a meaningful comparison for refgenie, which is more of a genomic data API and local file manager than a data storage system.*

2)

One nice additional feature would be to support the remote addition or update of genomes. For adoption beyond single research labs, this may prove to be an important feature because in many environments, the folks managing servers and those handling new genomes are likely to be in entirely different organisational units.

> *Do you mean that end users should be able to push new assets into a running server instance? This would be possible, but it would add some additional security and ownership concerns. Our approach was to instead make it easy for anyone to host their own server, and then make it easy to add additional genomes to a server instance. In fact, it would be relatively simple with the current system to automate server updates using a deployment strategy based on git hooks. By creating a list of genomes and assets to build for them as a git repository, users could add genome identifiers to a spreadsheet in this git repository, which would then be auto-deployed using travis to trigger a server update. The built-in archiving functionality of the server software (see `refgenieserver archive` command) provides an effortless way to add new servable archives. When new assets are built with `refgenie build`, execution of `refgenieserver archive` command respects the existent archives and just adds the newly created ones. Naturally, a subsequent execution of `refgenieserver serve` includes the recent additions. This makes the incremental updates to the servable data straightforward. So, in this environment, we would simply suggest that the "new genome managers" host a refgenie server instance, and then allow access to a git repository for any authorized user to add assets to the server. It seems that the security and storage issues that would be made possibly by allowing anonymous upload to the server from any source would be more hassle than the benefit is worth.*

3)

Along the same line, I am not sure I agree with the assertion "remote-hosted assets are only practical for common genomes and assets". As a group leader, I actually much prefer if the assets are centrally hosted than if they are scattered on the thumb drives of my lab members. . .

> *To us, these are two separate issues. Refgenie solves the scattered asset problem even without a refgenieserver instance. For the use case you describe, what we do is we have a single central local where we organize all of the lab's assets. We can there download or build whatever assets are needed by the lab. In fact, the new version of refgenie now includes improved provision for mutli-user access. Now, refgenie will lock the configuration file so that multiple users can use it simultaneously without worry of overwriting each others' edits. We recommend that a lab group set up a single refgenie genomes configuration file and archive, and then simply point the REFGENIE environment variable in everyone's environment to the same file. This way, an entire lab group can easily share a common set of genome assets. Not only that, but this organization would span research groups.*
>
> *The point of our statement is that it is not feasible for someone hosting a refgenieserver instance (my lab, for instance) to include every possible genome that everyone else could possibly want to use. Some subset of the assets we build could make sense to put into a refgenieserver instance, so that others can also use them; for these we could create a server. It wouldn't make sense to put everything in the server, because some of those assets are lab-specific or rarely used, so it's more practical for a local lab to just build them because only 1 or 2 labs need them. Otherwise, the refgenieserver would quickly require more resources than can be covered by a single lab. We don't expect to be able to download everything we need, since some things might be really specific to a particular analysis and only 3 labs in the world need those assets. In that case, we simply build them locally. So, there is no scatter; all of the lab's assets are locally hosted in a central location. This uses the asset management capabilities of the CLI. We would build whatever custom genomes we need.*
