# Supplementary material for: Refgenie: a reference genome resource manager
Source: Gigascience. 2020 Jan 29;9(2):giz149. doi: 10.1093/gigascience/giz149 (PMC6988606; doi:10.1093/gigascience/giz149)
Supplement: giz149_GIGA-D-19-00289_Revision_2 [file giz149_giga-d-19-00289_revision_2.pdf]

# GigaScience

## Refgenie: a reference genome resource manager

--Manuscript Draft--

| | |
|---|---|
| **Manuscript Number:** | GIGA-D-19-00289R2 |
| **Full Title:** | Refgenie: a reference genome resource manager |
| **Article Type:** | Technical Note |
| **Funding Information:** | National Institute of General Medical Sciences (1R35GM128636-01) — Dr. Nathan C. Sheffield |
| **Abstract:** | Reference genome assemblies are essential for high-throughput sequencing analysis projects. Typically, genome assemblies are stored on disk alongside related resources; for example, many sequence aligners require the assembly to be indexed. The resulting indexes are broadly applicable for downstream analysis, so it makes sense to share them. However, there is no simple tool to do this. To this end, we introduce refgenie, a reference genome assembly asset manager. Refgenie makes it easier to organize, retrieve, and share genome analysis resources. In addition to genome indexes, refgenie can manage any files related to reference genomes, including sequences and annotation files. Refgenie includes a command-line interface and a server application that provides a RESTful API, so it is useful for both tool development and analysis. Availability: https://refgenie.databio.org |
| **Corresponding Author:** | Nathan C. Sheffield<br><br>UNITED STATES |
| **Corresponding Author Secondary Information:** | |
| **Corresponding Author's Institution:** | |
| **Corresponding Author's Secondary Institution:** | |
| **First Author:** | Michał Stolarczyk |
| **First Author Secondary Information:** | |
| **Order of Authors:** | Michał Stolarczyk<br>Vincent Reuter<br>Jason P. Smith<br>Neal Magee<br>Nathan C. Sheffield |
| **Order of Authors Secondary Information:** | |
| **Response to Reviewers:** | Dear Hans,<br><br>Thanks for the positive response. We've now made all the requested changes suggested by reviewer 3. To respond to the question about building assets, we've put together more detailed documentation now here: http://refgenie.databio.org/en/latest/build/<br><br>We are considering ways to do the suggestion on cryptography, as suggested. I also found another similar tool ("genomepy") and added it to the tool comparison section. I added the GigaDB link as requested, and also reformatted the availability and requirements section as requested.<br><br>-Nathan |
| **Additional Information:** | |

Powered by Editorial Manager® and ProduXion Manager® from Aries Systems Corporation

| Question | Response |
| --- | --- |
| Are you submitting this manuscript to a special series or article collection? | No |
| **Experimental design and statistics**<br><br>Full details of the experimental design and statistical methods used should be given in the Methods section, as detailed in our Minimum Standards Reporting Checklist. Information essential to interpreting the data presented should be made available in the figure legends.<br><br>Have you included all the information requested in your manuscript? | No |
| If not, please give reasons for any omissions below.<br><br>as follow-up to "**Experimental design and statistics**<br><br>Full details of the experimental design and statistical methods used should be given in the Methods section, as detailed in our Minimum Standards Reporting Checklist. Information essential to interpreting the data presented should be made available in the figure legends.<br><br>Have you included all the information requested in your manuscript?<br><br>" | No experiments performed. |
| **Resources**<br><br>A description of all resources used, including antibodies, cell lines, animals and software tools, with enough information to allow them to be uniquely identified, should be included in the Methods section. Authors are strongly encouraged to cite Research Resource Identifiers (RRIDs) for antibodies, model | Yes |

| | |
|---|---|
| organisms and tools, where possible.<br><br>Have you included the information requested as detailed in our Minimum Standards Reporting Checklist? | |
| **Availability of data and materials**<br><br>All datasets and code on which the conclusions of the paper rely must be either included in your submission or deposited in publicly available repositories (where available and ethically appropriate), referencing such data using a unique identifier in the references and in the “Availability of Data and Materials” section of your manuscript.<br><br>Have you have met the above requirement as detailed in our Minimum Standards Reporting Checklist? | Yes |

Powered by Editorial Manager® and ProduXion Manager® from Aries Systems Corporation

Manuscript

Click here to access/download;Manuscript;manuscript.pdf

Click here to view linked References

RESEARCH ARTICLE

# Refgenie: a reference genome resource manager

Michał Stolarczyk[1,*], Vincent P. Reuter[1,*], Jason P. Smith[1,4], Neal E. Magee[5], and Nathan C. Sheffield[1,2,3,4,✉]

[1]Center for Public Health Genomics, University of Virginia
[2]Department of Public Health Sciences, University of Virginia
[3]Department of Biomedical Engineering, University of Virginia
[4]Department of Biochemistry and Molecular Genetics, University of Virginia
[5]Research Computing, University of Virginia
[*]Contributed equally
✉ Correspondence: nsheffield@virginia.edu

Reference genome assemblies are essential for high-throughput sequencing analysis projects. Typically, genome assemblies are stored on disk alongside related resources; for example, many sequence aligners require the assembly to be *indexed*. The resulting indexes are broadly applicable for downstream analysis, so it makes sense to share them. However, there is no simple tool to do this. To this end, we introduce refgenie, a reference genome assembly asset manager. Refgenie makes it easier to organize, retrieve, and share genome analysis resources. In addition to genome indexes, refgenie can manage any files related to reference genomes, including sequences and annotation files. Refgenie includes a command-line interface and a server application that provides a RESTful API, so it is useful for both tool development and analysis.

**Availability:** `https://refgenie.databio.org`

## Background

Enormous effort goes into assembling and curating reference genomes[1–5]. These reference assemblies provide a common representation for comparing results and they form the basis for a wide range of downstream tools for sequence alignment and annotation. Many tools that rely on reference assemblies will produce independent resources that accompany an assembly. For instance, many aligners must *hash* the genome, creating *indexes* that are used to improve alignment performance[6–9].

Analytical pipelines typically rely on these aligners and their indexes for the initial steps of a data analysis. These assembly resources are typically shared among many pipelines, so it's common for a research group to organize them in a central folder to prevent duplication. In addition to saving disk space, centralization simplifies sharing software that uses a reference assembly because software can be written around a standard folder structure. However, this does not solve the problem of sharing genomic resources *between* research groups. Because each group may use a different strategy to identify shared genome resources, sharing tools across groups may require modifying them.

One solution to this problem is to have a web-accessible server where standard, organized reference assemblies are available for download. Indeed, this is exactly the goal of Illumina's *iGenomes* project, which provides "a collection of reference sequences and annotation files for commonly analyzed organisms"[10]. The iGenomes project has become a popular source of genome assets and has greatly simplified sharing analysis tools among research environments. However, this approach suffers from some fundamental drawbacks and leaves several challenges unsolved. First, the individual assets can only be downloaded in bulk, but what if a particular use case requires only a small subset of resources in a package? More important, building the resources is not scripted, so if the repository excludes a reference or resource of interest, there is no programmatic way to fill the gap. In these scenarios, users must manually build and organize genome assets individually, forfeiting the strength of standardization among groups.

To improve the ability to share interoperable reference genome assets, we have developed *refgenie*, which enables a more modular, customizable, and user-controlled approach to managing reference assembly resources. Like iGenomes, refgenie standardizes reference genome asset organization so software can be built around that organization. But unlike iGenomes, refgenie also automates the *building* of genome assets, so that an identical representation can be produced for any genome assembly. Furthermore, refgenie allows programmatic access to individual resources both remote and local, making it suitable for the next generation of self-contained pipelines.

Refgenie can organize any files that can be assigned to a particular reference genome assembly, which could include not only genome indexes, but other resource types

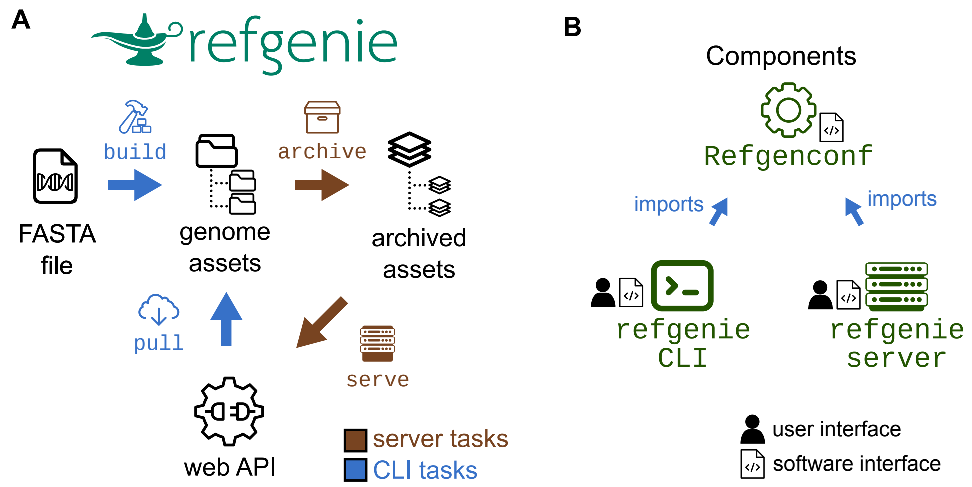


*Fig. 1: **Refgenie concept and software organization**. A: Refgenie provides the ability to either build or pull assets. B: Refgenie is tripartite, made up of a* `conf` *utility, a command-line interface (CLI), and a server package. The configuration package is intended for programmatic use, and is used by the CLI and server packages. Users and software use refgenie via the CLI or server (web API).*

like genome sequences and annotations[11–13].

Refgenie manages genome-related resources flexibly. It can handle any asset type, from annotations to indexes. It provides individual, pre-built asset downloads from a server and allows scripted building for custom inputs. Refgenie thus solves a major hurdle in biological data analysis.

## Results and discussion

Refgenie is the first full-service *reference genome asset manager*. Refgenie provides two ways to obtain genome assets: *pull*, and *build* (Fig.1A). For common assets, *pulling* a pre-built version obviates the need to install and run specialized software to build a particular asset. It also makes it easier to satisfy prerequisites programmatically for pipelines and other software. However, remote-hosted assets are only practical for common genomes and assets, so for uncommon assets or on unconnected computers, users may instead *build* assets, which creates the same standard output for custom genomes. By providing both *build* and *pull*, refgenie facilitates asset organization both within and between research groups, increasing interoperability of tools that rely on genome resources.

### Asset organization

Refgenie uses a local `YAML` file called the *genome configuration file* (Fig. 4) to keep track of metadata, such as local file paths. In this file, refgenie stores paths to individual genome assembly resources, or *assets*, each of which represents one or more files. You can think of a genome asset as a folder of related files tied to a particular genome assembly. For example, an asset could be an index for a particular tool, or a group of annotation files. Refgenie assets are referred to using *asset registry paths*, which are human-readable asset identifiers. The registry path follows the structure `{genome}/{asset}:{tag}`; a genome thus operates as a sort of namespace for a set of assets, which are identified both by asset names as well as tags, allowing refgenie to manage multiple versions of the same asset.

The `refgenie` software suite allows users to interact with assets with three components: 1) a command-line interface (CLI), 2) a server, and 3) a configuration package that supports them both (Fig.1B).

### Refgenie command-line interface

The workhorse of refgenie is the command-line interface (CLI); it is how users will typically interact with genome assets. Its implementation as a command-line tool not only makes it useful for general purpose exploration and access, but also allows it to be integrated into existing workflows that require access to genome assets from the shell. The CLI can be installed with `pip install refgenie` and invoked by calling `refgenie`. The refgenie CLI provides 7 functions for interacting with local genome assets:

- `refgenie init` – initializes an empty genome configuration file
- `refgenie list` – summarizes the genome configuration file, listing local genomes and assets
- `refgenie seek` – provides the file path to a given asset
- `refgenie add` – adds an already-built local asset
- `refgenie remove` – removes a local asset
- `refgenie tag` – adds a tag to a local asset
- `refgenie build` – builds a new asset

| asset name | genome | asset size | archive size | build time | peak memory |
|---|---|---|---|---|---|
| fasta | hg38 | 2.9 | 0.8 | 0:01 | <0.1 |
| bowtie2_index | hg38 | 3.9 | 3.5 | 0:57 | 5.6 |
| hisat2_index | hg38 | 4.2 | 3.9 | 0:36 | 5.5 |
| bismark_bt1_index | hg38 | 13.6 | 7.5 | 1:10 | 10.8 |
| bismark_bt2_index | hg38 | 13.6 | 7.5 | 2:17 | 10.8 |
| bwa_index | hg38 | 2.9 | 3.2 | 0:51 | 4.7 |
| star_index | hg38 | 26.9 | 24.3 | 1:51 | 35.8 |
| kallisto_index | hg38_cdna | 2.2 | 1.6 | 0:04 | 3.8 |
| salmon_index | hg38_cdna | 3.1 | 2.6 | 0:03 | 5.3 |
| dbnsfp | hg38 | 22.9 | 22.8 | 2:35 | *<0.1 |
| ensembl_rb | hg38 | <0.1 | <0.1 | 0:00 | <0.1 |
| ensembl_gtf | hg38 | <0.1 | <0.1 | 0:00 | <0.1 |
| gencode_gtf | hg38 | <0.1 | <0.1 | 0:00 | <0.1 |
| feat_annotation | hg38 | <0.1 | <0.1 | 0:01 | 0.1 |
| refgene_anno | hg38 | <0.1 | <0.1 | 0:00 | 0.2 |

*Fig.2: **Assets available for build**. Table listing assets that can currently be built with `refgenie build`, along with statistics for size, build time, and memory high water mark. Assets were built for the human genome using a single core. Times (in H:MM) and memory/disk (in gigabytes) are representative values from a single run. These assets are produced by various tools[8,9,14–17] and are available to be built for any arbitrary genome input. * peak disk space usage for dbnsfp is over 300GB*

### Initializing refgenie

All of the CLI commands require knowledge of the refgenie configuration file, which is passed via the `-c` argument. To install and configure refgenie requires only a few lines of code:

```
pip install --user refgenie
export REFGENIE="refgenie.yaml"
refgenie init -c $REFGENIE
```

In this example, we populate the `$REFGENIE` environment variable, which eliminates the need to pass `-c` to each command going forward. The `init`, `list`, `add`, and `remove` functions are relatively straightforward and simply allow a user to create, view, and manipulate the genome configuration file.

### Building assets

The `build` function allows a user to *build* assets for any arbitrary inputs, which is what enables refgenie to serve custom genomes. Refgenie has built-in capability to build a selection of different common genome assets (Fig.2). The list of assets with available recipes are listed by the `refgenie list` command. Available assets are built by specifying the asset registry path along with any required inputs. For example:

```
refgenie build hg38/ASSET \
  --INPUT FILE
```

Where `ASSET` is a unique key defining the asset of interest (*e.g.*, `bowtie2_index`), `INPUT` is an identifier for a required input, and `FILE` is a path to the provided input. For example, to build a `fasta` asset requires a compressed fasta file as input. It can thus be built like this:

```
refgenie build hg38/fasta \
  --fasta hg38.fa.gz
```

Building an asset can require either input arguments, such as in this example, or it can require other assets. The list of requirements for building an asset can be found by adding the `-r` argument to the `build` function. Assets are built with locally available versions of the software (*e.g.* `bowtie2-build` to create the Bowtie2 index) or alternatively with containerized software (using `-d/--docker` flag in the `refgenie build` command). We have also produced a complete containerized computing environment capable of building all available refgenie assets, which can be deployed with the *bulker* environment manager[18], making it easy to build any refgenie assets without installing the required tools natively.

### Pulling assets

In addition to functions on local assets, the refgenie CLI also contains additional commands that can interact with remote assets: *pull* and *listr*:

- `refgenie listr` – lists available remote genomes and assets
- `refgenie pull` – downloads a remote asset

With these commands, refgenie downloads a standard asset with a single line of code:

```
refgenie pull hg38/ASSET
```

### Tagging assets

The `tag` command allows users to tag assets with unique identifiers. Tags may also be provided when building or pulling assets to specify a version (*e.g.* `build hg38/ASSET:TAG`). Once tagged, specific versions of

assets can be accessed by tag. If no tag is specified, refgenie will use the tag `default`, which is automatically given to any built or pulled assets that do not specify a tag. This makes tags an optional feature of refgenie which are only necessary if a user desires multiple versions of the same asset.

### Seeking assets

Once the asset has been added to refgenie either via pulling or building, the user can retrieve the path to it with `refgenie seek`:

```
refgenie seek hg38/ASSET
```

This command returns the file path to the specified asset for the specified genome. The `seek` command is portable, eliminating the need to hard-code paths or pass them as arguments. Consequently, in a pipeline or other software that requires access to genome assembly assets, the path to the local `bowtie2_index` asset can be retrieved with a shell command:

```
bowtie2_index_path=\
  $(refgenie seek hg38/bowtie2_index)
```

## Refgenie server

The `listr` and `pull` functions require that the CLI interact with a server. The CLI uses a configurable URL to retrieve a remote archived tarball. After retrieving the tarball, the CLI will unpack it into the appropriate folder location and update the configuration file to provide access to its path via `refgenie seek`.

To support this remote function, we have developed a containerized, portable, open-source companion application called `refgenieserver`. Many users of refgenie will not have to be aware of the server application; however, interested users can use refgenie server to host their own genome asset server. For example, a tool developer may wish to simplify use by hosting indexes for common reference assemblies.

Running the refgenie server is simple for users who are already familiar with refgenie. It reads the same genome configuration file format as the CLI. In fact, refgenie server operates on the same genome configuration file and asset folders that that refgenie itself builds or downloads. The server software comes with an `archive` command that prepares a refgenie genome folder for serving. It compresses each asset into an individual tarball. This simple system makes it easy for users to run a server using their refgenie assets.

This server software leverages cutting-edge web technology to provide high-concurrency service with minimal compute resources (Fig. 3). We built refgenie server on top of the FastAPI Python framework, which is a high performance web framework for building APIs. FastAPI automatically produces an API that complies with OpenAPI 3.0 standards, which will allow other tools to discover and automatically use the API. It also includes a self-documenting test interface so that users can see and test the available API endpoints. Refgenie leverages the Starlette development toolkit and the `uvicorn` server to make use of the high-performance Asynchronous Server Gateway Interface (ASGI) specification, which provides asynchronous access to refgenie server.

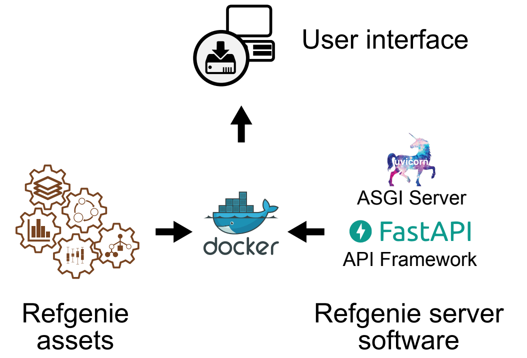


*Fig.3:* ***Server software stack.*** *Archived refgenie assets are mounted into a Docker container, along with the refgenie server software, which is built using FastAPI and uvicorn. The container can then be accessed via the web and API user interfaces.*

```
genome_folder: /genomes/path
genome_server: http://...
config_version: 0.3
genomes:
  hg38:
    assets:
      bowtie2_index:
        asset_description: ...
        default_tag: default
        tags:
          default:
            asset_path: bowtie2_index
            asset_digest: 0f9217d44264ae2188
            seek_keys:
              bowtie2_index: .
```

*Fig.4:* ***Genome configuration file.*** *Refgenie reads and writes a genome configuration file in YAML format to keep track of available local assets.*

Refgenie server is containerized and available on dockerhub, so that an interested user could run a server with a single line of code:

```
docker run --rm -p 80:80 \
  -v genomes_folder:/genomes rgimage \
   refgenie -c /genomes/config.yaml serve
```

By mounting a refgenie 'genomes' folder into this container, users get a fully functioning web interface and RESTful API.

### Refgenconf package for genome configuration

Refgenie organizes assets by genome in the configuration file, which is both computer-readable and human-readable. In practice, users will not need to interact with this file at all, as refgenie will handle both reading and writing the file. However, users may edit the file if they need a more complicated structure (such as storing assets on different file systems, or adding assets manually). Together with the refgenie software, this simple file makes the concept of reference genome assets completely portable. Full documentation for the configuration file format can be found at refgenie.databio.org.

The configuration package, `refgenconf`, simply provides functions and data types to read and write items listed in the genome configuration file. Under the hood, the refgenie CLI itself uses `refgenconf` to interact with the genome configuration and assets on disk. The server software also relies on it to read, archive, and serve assets. The `refgenconf` package also provides the starting point for any third-party Python developers by providing a fully functional Python application programming interface (API) for interacting with refgenie assets. For example, we use `refgenconf` in Python pipelines we develop to make them aware of the genome assets available in a given computing environment. Using this approach, a pipeline need only be provided with an assembly key, like 'hg38', and it can use `refgenconf` to locate the correct path to any genome-related asset necessary for the pipeline. This simplifies the process of configuring pipelines and allows `refgenie` to be used both by humans and computers.

### The Refgenomes database

We designed the server software so that anyone could easily run a custom server instance. We have also deployed our own instance of `refgenieserver` at refgenomes.databio.org, where we host pre-built genome assets. Like any instance of `refgenieserver`, our refgenomes database provides both a web interface and a RESTful API to access individual assets we have made available. Users may explore and download archived indexes from the web interface or develop tools that programmatically query the API.

The web interface provides a graphical listing of available genomes and assets, allowing users to browse the site and download individual assets manually. In addition, refgenieserver provides API endpoints to serve lists of available genomes and assets, as well as metadata for the individual assets, including unique digests for file integrity, file sizes, and archive content information. Furthermore, the server provides endpoints to download each asset individually. Endpoints include the following: `/genomes` retrieves a list of available genomes; `/assets` retrieves a list of all available assets; `/{genome}/assets/` retrieves a list of assets for a given genome; and `/{genome}/assets/{asset}/archive` retrieves the tarball for the specified asset. Complete documentation is available at refgenomes.databio.org. Because it provides a standard OpenAPI-compliant RESTful API, our server will be useful not just for our refgenie CLI, but for other tools that would benefit from automated access to reference assembly assets and indexes.

Our `refgenieserver` instance runs within DC/OS as a containerized application. The server application makes genome assets available through a web application connected directly to a remote filesystem, with no additional database or infrastructure requirements. Integration and deployment is automated using GitHub, Travis-CI, Docker Hub, and a custom deployment technique made simple in DC/OS. Changes committed in code are generally deployed to development or production services within 1-3 minutes.

### Genome provenance

One challenge with genome assembly assets is name mismatches that lead to analysis conflicts. Because refgenie identifiers are human-readable and user-controlled, refgenie cannot rely on them to uniquely identify assets. Furthermore, refgenie assets may be either built or pulled from different servers, exacerbating the issue. This is an active area of research, with several approaches under development related to this problem, such as the NCBI Assembly database[4], the refget protocol for sequence identifiers[19], and tximeta checksums for RNA-seq data[20]. Refgenie currently provides two resources to confirm the identity of pulled and built assets: First, a unique digest for each asset, and second, a building log file. Refgenie makes unique asset digests available via both web interface and API, allowing users to distinguish between two assets with the same names but different digests. Furthermore, because building refgenie assets is scripted, it is possible to trace any asset back to its inputs. Refgenie server provides API points to retrieve either the raw recipe (`/v2/asset/{genome}/{asset}/recipe`) or the actual log file (`/v2/asset/{genome}/{asset}/log`) for any asset available on the server. For built assets, the `build` command automatically produces a log file that records the input files, software versions, and final digests for any locally built assets. These resources make it possible for users to uniquely identify and trace the provenance of assets they either build or pull.

## Comparison to existing tools

A few existing tools approach these problems as well. The most similar projects are Illumina's *iGenomes* and Galaxy Data Managers accompanied by Galaxy Tool Shed[21,22], both of which offer only a small part of what refgenie does (Fig. 5). iGenomes provides a single

| | web interface to download assets | modular access to individual assets | custom genomes | RESTful API for assets | command-line interface and asset manager | portable command-line access to asset paths | containerized server software | programmatic API |
|---|---|---|---|---|---|---|---|---|
| Refgenie | ✓ | ✓ | ✓ | ✓ | ✓ | ✓ | ✓ | ✓ |
| iGenomes | ✓ | × | × | × | × | × | × | × |
| Data managers | ✓ | ✓* | ✓ | × | × | × | × | × |
| genomepy | × | ✓ | × | × | ✓ | × | × | ✓ |

*Fig.5*: ***Feature comparison***. *iGenomes, Galaxy Data managers, and genomepy solve some problems of standardized reference genome assets, but lack the interactive features of refgenie. *Data managers assets can be accessed individually, but not outside of the Galaxy UI.*

archive download of a standardized folder structure with pre-built assets for pre-defined genomes. The Data Managers facilitate building of assets, they are tightly coupled to the larger galaxy infrastructure, while Refgenie's modular design allows for simple implementation in diverse environments. The genomepy tool provides a unified command-line interface and python API to download genome sequences from multiple sources, but does not accommodate custom genomes and has no remote API or component for downloading indexes[23]. Some of refgenie's utility is also satisfied by individual tool websites that provide individual asset downloads (*e.g.* bowtie2 indexes), but these provide no shared structure or unified interface for access.

Refgenie provides a full-service manager that unifies and transcends all of these available tools. Refgenie solves a series of related problems all in one convenient package. It provides a unified web interface for all assets, plus programmatic access to modular individual assets via a RESTful API for metadata and assets. Refgenie also provides the ability to build assets for custom genomes with a uniform interface that integrates seamlessly with downloaded assets. Refgenie is unique in providing a local asset manager that makes locating assets portable, simplifying building pipelines that use these assets. It is also the only easily deployable, independent, containerized server application and Python API for reference genome assets. Thus, no existing software can solve these problems specific to genome-related data resources.

## Conclusions and future directions

Reference genomes, indexes, annotations, and other genome assets are integral to sequencing analysis projects, and these genome-associated data resources are growing rapidly[11]. Refgenie provides a full-service management system that includes a convenient method for downloading, building, sharing, and using these resources. Refgenieserver is among a growing number of API-oriented projects in the life sciences[5,24,25]. Refgenie will simplify management of reference assembly assets for users and groups, facilitating data sharing and software interoperability[26].

Several new features under development will make refgenie even more useful. Currently, refgenie is completely flexible with respect to genomes, but it is less flexible with respect to assets, as only pre-scripted assets can be built. A more flexible approach would allow refgenie to accept custom recipes, allowing users to add new asset types. Future development will address the challenges of sharing recipes, provenance, and trust for flexible assets. We are also improving the way refgenie records and uses identifiers and relationships among assets. For instance, by recording more detailed information about what an asset contains and how it was generated, we open the possibility of delineating more fine-grained compatibilities. For instance, while two indexes would only be compatible if derived from the same set of sequences, two annotation files could be compatible on different sequences that shared a coordinate structure. Finally, we anticipate that future development will extend refgenie to be able to accommodate ontology annotation for assets and genomes. Together, these improvements will enable more robust discovery of assets and genomes as well as the relationships among them.

## Availability and requirements

Project name: Refgenie
Project home page: http://refgenie.databio.org
Operating system: Platform independent
Programming language: Python
Other requirements: Varies by use case
License: BSD-2
RRID: SCR_017574
biotools ID: Refgenie

An archival copy of the code is available via the GigaScience database GigaDB[27].

## References

1. Harrow, J. *et al.* GENCODE: The reference human genome annotation for the ENCODE project. *Genome Research* **22,** 1760–1774 (2012).

2. Pruitt, K. D., Tatusova, T., Brown, G. R. & Maglott, D. R. NCBI reference sequences (RefSeq): Current status, new features and genome annotation policy. *Nucleic Acids Research* **40,** D130–D135 (2011).

3. Church, D. M. *et al.* Modernizing reference genome assemblies. *PLoS Biology* **9,** e1001091 (2011).

4. Kitts, P. A. *et al.* Assembly: A resource for assembled genomes at NCBI. *Nucleic Acids Research* **44,** D73–D80 (2015).

5. Ruffier, M. *et al.* Ensembl core software resources: Storage and programmatic access for DNA sequence and genome annotation. *Database* **2017,** (2017).

6. Sadakane, K. & Shibuya, T. Indexing huge genome sequences for solving various problems. *Genome Informatics* **12,** 175–183 (2001).

7. Hon, W.-K., Sadakane, K. & Sung, W.-K. Breaking a time-and-space barrier in constructing full-text indices. *SIAM Journal on Computing* **38,** 2162–2178 (2009).

8. Li, H. & Durbin, R. Fast and accurate short read alignment with burrows-wheeler transform. *Bioinformatics* **25,** 1754–60 (2009).

9. Langmead, B. & Salzberg, S. L. Fast gapped-read alignment with bowtie 2. *Nat. Methods* **9,** 357–359 (2012).

10. Illumina. IGenomes. Ready-to-use reference sequences and annotations. *support.illumina.com* (2019).

11. Richa Agarwala *et al.* Database resources of the national center for biotechnology information. *Nucleic Acids Research* **46,** D8–D13 (2018).

12. Zerbino, D. R., Wilder, S. P., Johnson, N., Juettemann, T. & Flicek, P. R. The Ensembl Regulatory Build. *Genome Biology* **16,** (2015).

13. Sheffield, N. C. & Bock, C. LOLA: Enrichment analysis for genomic region sets and regulatory elements in R and bioconductor. *Bioinformatics* **32,** 587–589 (2016).

14. Krueger, F. & Andrews, S. R. Bismark: A flexible aligner and methylation caller for bisulfite-seq applications. *Bioinformatics* **27,** 1571–1572 (2011).

15. Bray, N. L., Pimentel, H., Melsted, P. & Pachter, L. Near-optimal probabilistic RNA-seq quantification. *Nature Biotechnology* **34,** 525–527 (2016).

16. Kim, D., Langmead, B. & Salzberg, S. L. HISAT: A fast spliced aligner with low memory requirements. *Nature Methods* **12,** 357–360 (2015).

17. Dobin, A. *et al.* STAR: Ultrafast universal RNA-seq aligner. *Bioinformatics* **29,** 15–21 (2012).

18. Sheffield, N. C. Bulker: A multi-container environment manager. *OSF Preprints* (2019). doi:10.31219/osf.io/natsj

19. GA4GH. Refget - reference sequence retrieval implementation. *samtools.github.io/* (2019).

20. Love, M. I. *et al.* Tximeta: Reference sequence checksums for provenance identification in RNA-seq. *bioRxiv* (2019). doi:10.1101/777888

21. Blankenberg, D., Johnson, J. E., Taylor, J. & Nekrutenko, A. Wrangling galaxy's reference data. *Bioinformatics* **30,** 1917–1919 (2014).

22. Blankenberg, D. *et al.* Dissemination of scientific software with galaxy ToolShed. *Genome Biology* **15,** 403 (2014).

23. Heeringen, S. J. van. Genomepy: Download genomes the easy way. *The Journal of Open Source Software* **2,** 320 (2017).

24. Yates, A. *et al.* The ensembl REST API: Ensembl data for any language. *Bioinformatics* **31,** 143–145 (2014).

25. Tarkowska, A. *et al.* Eleven quick tips to build a usable REST API for life sciences. *PLOS Computational Biology* **14,** e1006542 (2018).

26. Wilkinson, M. D. *et al.* The FAIR guiding principles for scientific data management and stewardship. *Sci. Data* **3,** 160018 (2016).

27. Stolarczyk, M., Reuter, V. P., Smith, J. P., Magee, N. E. & Sheffield, N. C. Supporting data for "refgenie: A reference genome resource manager". *GigaScience Database* (2019). doi:10.5524/100670
