# Supplementary material for: Refgenie: a reference genome resource manager
Source: Gigascience. 2020 Jan 29;9(2):giz149. doi: 10.1093/gigascience/giz149 (PMC6988606; doi:10.1093/gigascience/giz149)
Supplement: giz149_Response_to_Reviewer_Comments_Original_Submission [file giz149_response_to_reviewer_comments_original_submission.pdf]

Author's Response To Reviewer Comments

Close

Response to review is included as a PDF cover letter for ease of review.

Close
