# Supplementary material for: Refgenie: a reference genome resource manager
Source: Gigascience. 2020 Jan 29;9(2):giz149. doi: 10.1093/gigascience/giz149 (PMC6988606; doi:10.1093/gigascience/giz149)
Supplement: giz149_Response_to_Reviewer_Comments_Revision_1 [file giz149_response_to_reviewer_comments_revision_1.pdf]

Author's Response To Reviewer Comments

Close

Dear Hans,

Thanks for the positive response. We've now made all the requested changes suggested by reviewer 3. To respond to the question about building assets, we've put together more detailed documentation now here: http://refgenie.databio.org/en/latest/build/

We are considering ways to do the suggestion on cryptography, as suggested. I also found another similar tool ("genomepy") and added it to the tool comparison section. I added the GigaDB link as requested, and also reformatted the availability and requirements section as requested.

-Nathan

Close
