# Supplementary material for: Refgenie: a reference genome resource manager
Source: Gigascience. 2020 Jan 29;9(2):giz149. doi: 10.1093/gigascience/giz149 (PMC6988606; doi:10.1093/gigascience/giz149)
Supplement: giz149_Reviewer_1_Report_Original_Submission — Andrew Yates -- 8/14/2019 Reviewed [file giz149_reviewer_1_report_original_submission.pdf]

Reviewer Report

Title: Refgenie: a reference genome resource manager

Version: Original Submission **Date:** 8/14/2019

Reviewer name: Andrew Yates

Reviewer Comments to Author:

Title: Refgenie: a reference genome resource manager
Recommendation: Accept with possible minor modifications
Key Questions
Does the manuscript adhere to the journal's guidelines on minimum standards of reporting?
Yes
Are methods appropriate?
Yes
Are the conclusions adequately supported by the data shown?
Yes
Quality of language?
High and requires no additional editing
Are you able to assess all statistics in the manuscript, including the appropriateness of statistical tests used?
No statistics are given in the manuscript so there is no need to assess this.
Review
The manuscript describes a single tool for the management and distribution of genome related assets for use in downstream analysis, such as variant calling and RNA-seq quantification. Refgenie is both a python binary for assets management on a local system and a web API for centralising and disseminating said assets, which can be used independently of the refgenie binary. The tool is both novel and useful capable of an immediate impact within any analysis workflow. As such I believe the manuscript should be accepted and raise only a set of minor points detailed below.
1). Comparison of refgenie to iGenomes
As stated in the manuscript, the iGenomes resource is one of the few resources which attempts to provide similar functionality. One aspect missed out of the comparison between the two is that iGenomes covers a much wider number of genomes and annotation sets than the reference deployment at refgenomes.databio.org.
Documentation at refgenie.databio.org has information on how a researcher could host iGenomes data within refgenie but would the authors be open to mirroring the entire contents of iGenomes on their reference implementation or to create a standard import module to help users import an entire iGenomes file in a single command. I feel that would help drive adoption and remove the last point here iGenomes still has an upper hand over refgenie.
2). Lack of future directions and detailing of the limitations of the current refgenie implementation
The manuscript does not detail the areas and enhancements refgenie would look towards implementing

in the future. This for me would include a). how to easily inject new asset creation methods into the build process e.g. if a new tool appeared that gained popularity how would that be supported b). steps refgenie will take to ensure portability of genome identifiers across resources and ensuring that these resources are the same.
On this second point I should disclose that the corresponding author, Nathan Sheffield, has recently joined a Global Alliance for Genomics and Health project called refget, which I head up. I know that the work he is participating on will help to resolve the issue of genome identity across resources. Whilst I am not looking for a namecheck here, I do feel the authors should flag this as a potential issue and that steps are being taken to address it.
Overall though the manuscript is excellent and I congratulate the authors on their work.
Andrew Yates
EMBL-EBI

Level of Interest

Please indicate how interesting you found the manuscript: Choose an item.

Quality of Written English

Please indicate the quality of language in the manuscript: Choose an item.

Declaration of Competing Interests

Please complete a declaration of competing interests, considering the following questions:

- Have you in the past five years received reimbursements, fees, funding, or salary from an organisation that may in any way gain or lose financially from the publication of this manuscript, either now or in the future?
- Do you hold any stocks or shares in an organisation that may in any way gain or lose financially from the publication of this manuscript, either now or in the future?
- Do you hold or are you currently applying for any patents relating to the content of the manuscript?
- Have you received reimbursements, fees, funding, or salary from an organization that holds or has applied for patents relating to the content of the manuscript?
- Do you have any other financial competing interests?
- Do you have any non-financial competing interests in relation to this paper?

If you can answer no to all of the above, write 'I declare that I have no competing interests' below. If your reply is yes to any, please give details below.

Yes. I am the head of a global alliance for genomics and health work stream on a product called refget. Nathan Sheffield has recently joined this effort and is contributing partly due to his with reference genome datasets and refgenie.

I agree to the open peer review policy of the journal. I understand that my name will be included on my report to the authors and, if the manuscript is accepted for publication, my named report including any attachments I upload will be posted on the website along with the authors' responses. I agree for my report to be made available under an Open Access Creative Commons CC-BY license (http://creativecommons.org/licenses/by/4.0/). I understand that any comments which I do not wish to be included in my named report can be included as confidential comments to the editors, which will not be published.

Choose an item.

To further support our reviewers, we have joined with Publons, where you can gain additional credit to further highlight your hard work (see: https://publons.com/journal/530/gigascience). On publication of this paper, your review will be automatically added to Publons, you can then choose whether or not to claim your Publons credit. I understand this statement.

Yes Choose an item.
