# Supplementary material for: Refgenie: a reference genome resource manager
Source: Gigascience. 2020 Jan 29;9(2):giz149. doi: 10.1093/gigascience/giz149 (PMC6988606; doi:10.1093/gigascience/giz149)
Supplement: giz149_Reviewer_2_Report_Original_Submission — Katherine James -- 8/26/2019 Reviewed [file giz149_reviewer_2_report_original_submission.pdf]

Reviewer Report

Title: Refgenie: a reference genome resource manager

Version: Original Submission **Date:** 8/26/2019

Reviewer name: Katherine James

Reviewer Comments to Author:

Refgenie is a very useful tool that has several functions that are not provided by iGenomes. In particular, it allows the use of custom genomes, which will become increasingly necessary as researchers work with more diverse species following recent large genome sequencing projects, such as the International Vertebrate Sequencing Project. Refgenie would also be of particular use to groups working with multiple bacterial strains, where a large number of reference genomes may be in use.

The manuscript is extremely well written and describes the architecture and usage of Refgenie. I have four minor comments:

1. The results section introduces the three components of Refgenie: 1) command-line interface, 2) server, 3) configuration package but discusses them in the following subsections in a different order, and after the 'Genome configuration and asset organisation' section. From the readers point of view, reordering these sections to describe these three components in this order would aid clarity. Indeed, the 'command-line interface' section is likely to be of most interest to potential users of Refgenie. Additionally, as a suggestion only, combining the 'Genome configuration and asset organisation' with 'Refgenconf configuration package' would also aid clarity.
2. The 'command line interface' section provides some example commands for a download of a bowtie2 index for hg38. For better understanding of refgenie usage, it would be useful if it also had similar real world examples for the other local genome assets commands, for instance a build for a local resource.
3. In the same section, a real example of how the refgenie seek command is portable and eliminates the need for hard coded paths would aid understanding of this concept.
4. In the section, 'comparison with existing tools' the authors state that iGenomes is only available as a single archive download. However, it should be noted that there are other sources for download of indexed reference genomes. For instance pre-built indexes for model organisms are available individually from the bowtie2 website and via the command line from the linked ftp site. Additionally, the Galaxy Tool Shed provides data managers for index builds for multiple tools. This section should be expanded to highlight the unique features provided by Refgenie over existing resources for genome assets.

Level of Interest

Please indicate how interesting you found the manuscript: Choose an item.

Quality of Written English

Please indicate the quality of language in the manuscript: Choose an item.

Declaration of Competing Interests

Please complete a declaration of competing interests, considering the following questions:

- Have you in the past five years received reimbursements, fees, funding, or salary from an organisation that may in any way gain or lose financially from the publication of this manuscript, either now or in the future?
- Do you hold any stocks or shares in an organisation that may in any way gain or lose financially from the publication of this manuscript, either now or in the future?
- Do you hold or are you currently applying for any patents relating to the content of the manuscript?
- Have you received reimbursements, fees, funding, or salary from an organization that holds or has applied for patents relating to the content of the manuscript?
- Do you have any other financial competing interests?
- Do you have any non-financial competing interests in relation to this paper?

If you can answer no to all of the above, write 'I declare that I have no competing interests' below. If your reply is yes to any, please give details below.

I declare that I have no competing interests

I agree to the open peer review policy of the journal. I understand that my name will be included on my report to the authors and, if the manuscript is accepted for publication, my named report including any attachments I upload will be posted on the website along with the authors' responses. I agree for my report to be made available under an Open Access Creative Commons CC-BY license (http://creativecommons.org/licenses/by/4.0/). I understand that any comments which I do not wish to be included in my named report can be included as confidential comments to the editors, which will not be published.

Choose an item.

To further support our reviewers, we have joined with Publons, where you can gain additional credit to further highlight your hard work (see: https://publons.com/journal/530/gigascience). On publication of this paper, your review will be automatically added to Publons, you can then choose whether or not to claim your Publons credit. I understand this statement.

Yes Choose an item.
