# Supplementary material for: Refgenie: a reference genome resource manager
Source: Gigascience. 2020 Jan 29;9(2):giz149. doi: 10.1093/gigascience/giz149 (PMC6988606; doi:10.1093/gigascience/giz149)
Supplement: giz149_Reviewer_3_Report_Original_Submission — Bernie Pope, Ph.D. -- 8/29/2019 Reviewed [file giz149_reviewer_3_report_original_submission.pdf]

Reviewer Report

Title: Refgenie: a reference genome resource manager

Version: Original Submission **Date:** 8/29/2019

Reviewer name: Bernie Pope, Ph.D.

Reviewer Comments to Author:

This paper describes Refgenie a system for managing and distributing genome reference files and their associated assets (such as index files etc).

Managing genome references and their associated files is a common problem faced by bioinformaticians and Refgenie provides a potentially useful service in this area. Refgenie is based on a server system for storing and sharing reference data, and a command line interface for interacting with the server. A user can request a copy of an existing reference resource from the server, or if that does not already exist, they can supply a new FASTA file and ask Regenie to automatically create a new reference resource from that file.
Sharing and managing genome reference data can be useful within research groups and organisations, where consistency in analyses is important.
Overall this is a well written and tidy paper that describes a potentially useful tool that may be of interest to the journal readership.
I have a few comments about the paper that I believe should be addressed before publication:
1) An important issue with sharing and reusing genome assets is provenance and trust. I can foresee that users will want some level of certification about who has supplied the genome and how it was processed. For instance, perhaps
genome assets could use public key cryptography to sign the data and provide a level of certainty about its origin? In short, how can we trust the data that we get back from Refgenie?
2) The authors suggest that there is little prior work for comparison, which is probably true. However, I think one possible partial competitor is the CVMFS system used by the Galaxy project, which has been used to share reference
data resources, e.g. https://training.galaxyproject.org/training-material/topics/admin/tutorials/cvmfs/tutorial.html. I think it is worth comparing Refgenie to CVMFS.
3) On page 3 I found it slightly difficult to understand how the build command works. Where does it run? How does it know what to run? Can the user control the versions of tools which are run?
4) I'm not sure that "lightning fast" is an appropriate adjective for a scientific paper.
5) On page 5 there is quite a lot of technical detail about the implementation of the refgenie server. I'm not sure that this level of detail is required for the paper.
6) Please use "Python" (proper noun) for the programming language instead of "python".
7) I tried to use Refgenie on the HPC system where I work. However, I ran into a problem:

```
$ python3 --version
Python 3.7.1
```

```
$ python3 -m venv refgenie_dev
$ source refgenie_dev/bin/activate
(refgenie_dev)$ refgenie
Traceback (most recent call last):
 File "/home/foo/scratch/refgenie_dev/bin/refgenie", line 11, in
   load_entry_point('refgenie==0.6.0', 'console_scripts', 'refgenie')()
 File "/home/foo/scratch/refgenie_dev/lib/python3.7/site-packages/refgenie/refgenie.py", line 387, in
main
   parser = logmuse.add_logging_options(build_argparser())
 File "/home/foo/scratch/refgenie_dev/lib/python3.7/site-packages/refgenie/refgenie.py", line 98, in
build_argparser
   sps[BUILD_CMD], groups=None, args=["recover", "config", "new-start"])
 File "/home/foo/scratch/refgenie_dev/lib/python3.7/site-packages/pypiper/utils.py", line 60, in
add_pypiper_args
   argument_groups=groups, arguments=args, use_all_args=all_args)
 File "/home/foo/scratch/refgenie_dev/lib/python3.7/site-packages/pypiper/utils.py", line 808, in
_determine_args
   from logmuse import LOGGING_CLI_OPTDATA
```

I tried other versions of Python 3, but still had the same problem.

Level of Interest

Please indicate how interesting you found the manuscript: Choose an item.

Quality of Written English

Please indicate the quality of language in the manuscript: Choose an item.

Declaration of Competing Interests

Please complete a declaration of competing interests, considering the following questions:

- Have you in the past five years received reimbursements, fees, funding, or salary from an organisation that may in any way gain or lose financially from the publication of this manuscript, either now or in the future?
- Do you hold any stocks or shares in an organisation that may in any way gain or lose financially from the publication of this manuscript, either now or in the future?
- Do you hold or are you currently applying for any patents relating to the content of the manuscript?
- Have you received reimbursements, fees, funding, or salary from an organization that holds or has applied for patents relating to the content of the manuscript?
- Do you have any other financial competing interests?

- Do you have any non-financial competing interests in relation to this paper?

If you can answer no to all of the above, write 'I declare that I have no competing interests' below. If your reply is yes to any, please give details below.

I declare that I have no competing interests

I agree to the open peer review policy of the journal. I understand that my name will be included on my report to the authors and, if the manuscript is accepted for publication, my named report including any attachments I upload will be posted on the website along with the authors' responses. I agree for my report to be made available under an Open Access Creative Commons CC-BY license (http://creativecommons.org/licenses/by/4.0/). I understand that any comments which I do not wish to be included in my named report can be included as confidential comments to the editors, which will not be published.

Choose an item.

To further support our reviewers, we have joined with Publons, where you can gain additional credit to further highlight your hard work (see: https://publons.com/journal/530/gigascience). On publication of this paper, your review will be automatically added to Publons, you can then choose whether or not to claim your Publons credit. I understand this statement.

Yes Choose an item.
