# Supplementary material for: Refgenie: a reference genome resource manager
Source: Gigascience. 2020 Jan 29;9(2):giz149. doi: 10.1093/gigascience/giz149 (PMC6988606; doi:10.1093/gigascience/giz149)
Supplement: giz149_Reviewer_3_Report_Revision_1 — Bernie Pope, Ph.D. -- 10/24/2019 Reviewed [file giz149_reviewer_3_report_revision_1.pdf]

Reviewer Report

Title: Refgenie: a reference genome resource manager

Version: Revision 1 **Date:** 10/24/2019

Reviewer name: Bernie Pope, Ph.D.

Reviewer Comments to Author:

I thank the authors for their careful consideration of the reviewer comments from the previous submission. I also commend the authors for the engineering efforts put into Refgenie.

I believe that all the major issues raised in the previous reviews have been addressed in the manuscript and the software, and am therefore happy to recommend this manuscript for publication.

The additional material regarding asset provenance is welcome, and I encourage the authors to consider some kind of "signing" mechanism (e.g. crypto based) to identify the source of the asset. The current mechanisms in the paper appear to solve the issue of telling different assets apart, however there still seems to be room for a malicious agent to provide untrustworthy data to be shared. If Refgenie becomes popular then it will likely become a more interesting target for such activity (unfortunately).

I think it might be too strong to say "Refgenie fills a niche for which, to our knowledge, there is no other competing software." I think there _are_ competing systems (the ones you mention in the paper). They may not be as feature-complete, but they are addressing broadly similar concerns, and therefore competing. Maybe this can be rephrased?

One detail that I am still not sure about is local building of assets. In the author's response it says that "The build process runs on the user's computer locally, so it uses whatever versions are available in the user's PATH". What happens if the asset depends on the version of the tool that was applied? For example, a tool (such as BWA) changes how indexes work between versions, and old indexes only work with old versions. Relying on building assets on the local machine seems like it might be fraught with version-compatibility issues?

Some minor suggestions for the final publication:

Fig 2 (should that be Table 2?): I would recommend removing "GB" from next to each of the numbers, and instead, put "(GB)" next to the respective column titles. It might also be best to align the numbers to the left of the columns, rather than the centre, to make it easier to read. Also, a peak memory usage of 0GB seems unusual. Surely some non zero amount of memory is needed? Perhaps this is rounded down, but maybe there is another way to show low memory usage (eg <1GB).

Page 4: I suggest changing "config file" with "configuration file".

Fig 5 (should that be Table X?): Perhaps do not focus on a "python" (should be "Python") API. It probably doesn't matter too much which language is used for the API, but is more significant that a programming API is available at all. Sure, Python is popular in bioniformatics, but other languages would equally be useful. So maybe just say "API".

Level of Interest

Please indicate how interesting you found the manuscript: Choose an item.

Quality of Written English

Please indicate the quality of language in the manuscript: Choose an item.

Declaration of Competing Interests

Please complete a declaration of competing interests, considering the following questions:

- Have you in the past five years received reimbursements, fees, funding, or salary from an organisation that may in any way gain or lose financially from the publication of this manuscript, either now or in the future?
- Do you hold any stocks or shares in an organisation that may in any way gain or lose financially from the publication of this manuscript, either now or in the future?
- Do you hold or are you currently applying for any patents relating to the content of the manuscript?
- Have you received reimbursements, fees, funding, or salary from an organization that holds or has applied for patents relating to the content of the manuscript?
- Do you have any other financial competing interests?
- Do you have any non-financial competing interests in relation to this paper?

If you can answer no to all of the above, write 'I declare that I have no competing interests' below. If your reply is yes to any, please give details below.

I declare that I have no competing interests

I agree to the open peer review policy of the journal. I understand that my name will be included on my report to the authors and, if the manuscript is accepted for publication, my named report including any attachments I upload will be posted on the website along with the authors' responses. I agree for my report to be made available under an Open Access Creative Commons CC-BY license (http://creativecommons.org/licenses/by/4.0/). I understand that any comments which I do not wish to be included in my named report can be included as confidential comments to the editors, which will not be published.

Choose an item.

To further support our reviewers, we have joined with Publons, where you can gain additional credit to further highlight your hard work (see: https://publons.com/journal/530/gigascience). On publication of this paper, your review will be automatically added to Publons, you can then choose whether or not to claim your Publons credit. I understand this statement.

Yes Choose an item.
