# Supplementary material for: Refgenie: a reference genome resource manager
Source: Gigascience. 2020 Jan 29;9(2):giz149. doi: 10.1093/gigascience/giz149 (PMC6988606; doi:10.1093/gigascience/giz149)
Supplement: giz149_Reviewer_4_Report_Original_Submission — Christophe Dessimoz -- 9/4/2019 Reviewed [file giz149_reviewer_4_report_original_submission.pdf]

Reviewer Report

Title: Refgenie: a reference genome resource manager

Version: Original Submission **Date:** 9/4/2019

Reviewer name: Christophe Dessimoz

Reviewer Comments to Author:

The submission present a system called "refgenie" to build, store, and share genomes and associated indices, which are often required by downstream processes. The authors argue that this is a unique niche, the closest related system being Illumina's iGenomes.
I agree that the practical problem tackled is of high interest, and that the software proposed is a promising solution to that problem. The manuscript is clearly written and nicely showcases the high-level features. This is complemented by extensive documentation and tutorials, neat code, and a highly user friendly installation procedure through PyPI.
In particular, the flexibility of supporting remote retrieval of data via CLI, API and a web interface makes refgenie a compelling solution.
I have no major or minor reservation, just a few discretionary points to the authors.
* One alternative would be to use git lfs for the storage of genomic data. This could be added to the comparison table (with appropriate amendements to the columns, if sensible)
* One nice additional feature would be to support the remote addition or update of genomes. For adoption beyond single research labs, this may prove to be an important feature because in many environments, the folks managing servers and those handling new genomes are likely to be in entirely different organisational units.
* Along the same line, I am not sure I agree with the assertion "remote-hosted assets are only practical for common genomes and assets". As a group leader, I actually much prefer if the assets are centrally hosted than if they are scattered on the thumb drives of my lab members...

Level of Interest

Please indicate how interesting you found the manuscript: Choose an item.

Quality of Written English

Please indicate the quality of language in the manuscript: Choose an item.

Declaration of Competing Interests

Please complete a declaration of competing interests, considering the following questions:

- Have you in the past five years received reimbursements, fees, funding, or salary from an organisation that may in any way gain or lose financially from the publication of this manuscript, either now or in the future?
- Do you hold any stocks or shares in an organisation that may in any way gain or lose financially from the publication of this manuscript, either now or in the future?
- Do you hold or are you currently applying for any patents relating to the content of the manuscript?
- Have you received reimbursements, fees, funding, or salary from an organization that holds or has applied for patents relating to the content of the manuscript?
- Do you have any other financial competing interests?
- Do you have any non-financial competing interests in relation to this paper?

If you can answer no to all of the above, write 'I declare that I have no competing interests' below. If your reply is yes to any, please give details below.

I have no competing interests.

I agree to the open peer review policy of the journal. I understand that my name will be included on my report to the authors and, if the manuscript is accepted for publication, my named report including any attachments I upload will be posted on the website along with the authors' responses. I agree for my report to be made available under an Open Access Creative Commons CC-BY license (http://creativecommons.org/licenses/by/4.0/). I understand that any comments which I do not wish to be included in my named report can be included as confidential comments to the editors, which will not be published.

Choose an item.

To further support our reviewers, we have joined with Publons, where you can gain additional credit to further highlight your hard work (see: https://publons.com/journal/530/gigascience). On publication of this paper, your review will be automatically added to Publons, you can then choose whether or not to claim your Publons credit. I understand this statement.

Yes Choose an item.
